# Conversion of self-contained breathing apparatus mask to open source powered air-purifying particulate respirator for fire fighter COVID-19 response

**DOI:** 10.1016/j.ohx.2020.e00129

**Published:** 2020-07-27

**Authors:** Benjamin R. Hubbard, Joshua M. Pearce

**Affiliations:** a Department of Mechanical Engineering-Engineering Mechanics, Michigan Technological University, Houghton, MI 49931, USA; b Department of Materials Science & Engineering, Michigan Technological University, USA; c Department of Electrical & Computer Engineering, Michigan Technological University, USA; d Équipe de Recherche sur les Processus Innovatifs (ERPI), Université de Lorraine, France; e School of Electrical Engineering, Aalto University, Finland

**Keywords:** Open hardware, COVID-19, Medical hardware, Powered Air-Purifying Respirator, PAPR, RepRap, 3-D printing, Additive manufacturing, Personal protective equipment, Safety equipment

## Abstract

To assist firefighters and other first responders to use their existing equipment for respiration during the COVID-19 pandemic without using single-use, low-supply, masks, this study outlines an open source kit to convert a 3M-manufactured Scott Safety self-contained breathing apparatus (SCBA) into a powered air-purifying particulate respirator (PAPR). The open source PAPR can be fabricated with a low-cost 3-D printer and widely available components for less than $150, replacing commercial conversion kits saving 85% or full-fledged proprietary PAPRs saving over 90%. The parametric designs allow for adaptation to other core components and can be custom fit specifically to fire-fighter equipment, including their suspenders. The open source PAPR has controllable air flow and its design enables breathing even if the fan is disconnected or if the battery dies. The open source PAPR was tested for air flow as a function of battery life and was found to meet NIOSH air flow requirements for 4 h, which is 300% over expected regular use.

## Graphical abstract

**Figure:**
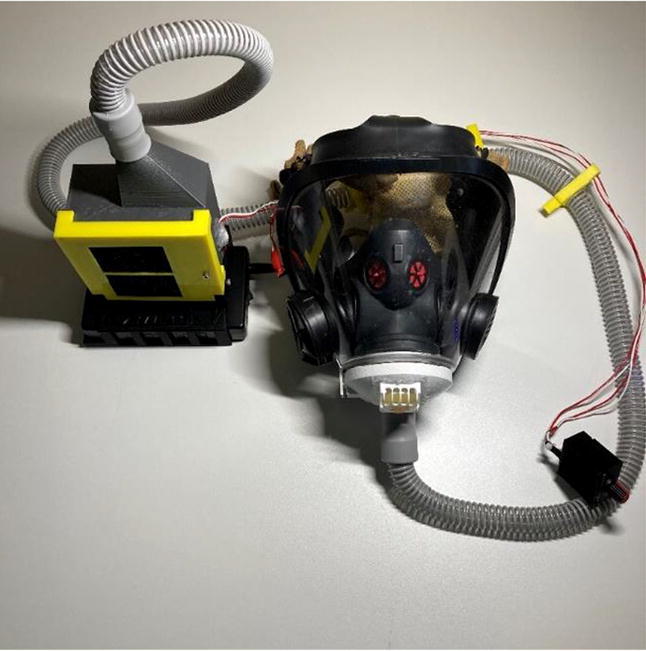

**Table:** 

Hardware name	Open Source Powered Air-Purifying Respirator
Subject area	• Medical
Hardware type	• Personal Protective Equipment
Open Source License	GNU General Public License (GPL) v3.0 and CERN Open Hardware License (OHL) v1.2
Cost of Hardware	$146
Source File Repository	Repository: https://osf.io/ydfmc/ Registration: https://osf.io/bdcjp

## 1 Hardware in context

Coronavirus disease 2019 (COVID-19) is challenging our medical infrastructure [1], [2], [3], [4] and has caused a critical shortage of personal protective equipment (PPE) [5], [6], [7], [8], [9]. As COVID-19 continues to spread through the highest-death-toll country, the U.S. [10], American first responders such as fire fighters on the frontlines, sometimes without appropriate PPE, are increasingly vulnerable to contracting the virus and the national death toll now includes a growing number of fire service personnel [11].

The White House has implemented the Defense Production Act [12] to force manufacturers to provide more PPE, but the administration has sown confusion concerning timing, PPE distribution and priorities [13]. PPE shortages thus persist at a regional and community level, so, to fill the gap, there has been a surge of distributed manufacturing of PPE [14], [15], [16], [17] using open hardware designs [18], [19], [20]. In this relatively new model, technically and economically viable open source small-scale digital technologies are used for distributed manufacturing [21], [22] by small-businesses [23], fab labs [24], [25], or even individuals at home [26], [27], [28], [29]. Open source medical equipment [30], [31], [32], [33] has already been shown to be particularly adept at overcoming supply shortages in a range of contexts [34], [35], [36], [37], [38]. The U.S. Centers for Disease Control and Prevention (CDC) recommends that first responders should where an N-95 or higher-level respirator or facemask (if a respirator is not available) [39]. To assist firefighters and other first responders meet this requirement, this study outlines an open source kit to convert a Scott Safety self-contained breathing apparatus (SCBA) into a powered air-purifying particulate respirator (PAPR). An SCBA uses compressed air, fed to an air-tight mask via a pressure regulator, while a PAPR uses filtered forced-air. SCBAs and PAPRs both provide clean air to the wearer, but using a SCBA for all tasks is unrealistic and cost-prohibitive. In this case, pre-existing equipment (the SCBA facepiece) will be coupled to a low-cost system to provide a PAPR to all fire departments or others that already have SCBAs. Those using the PAPRs will be able to interact with COVID-19 patients without putting themselves at heightened risk and without using low-supply, single-use PPE. This design couples with a Scott Safety AV3000 SCBA facepiece because the 3M manufactured SCBA is one of the most widespread on the market [40], however, this parametric design enables adaptation to other SCBAs facepieces with customization of a single 3-D printed part.

## 2 Hardware description

To overcome the current PPE shortage and allow anyone in the future to use a Scott Safety AV3000 SCBA facepiece [41] as a PAPR, this study develops a distributed manufacturing solution using only an open source manufacturing tool chain and provides a parametric fully free design of the components needed for the conversion. Following well-established open hardware design protocols [18], [19], [20], the PAPR was designed using parametric FreeCAD [42].

**Fig. 1:**
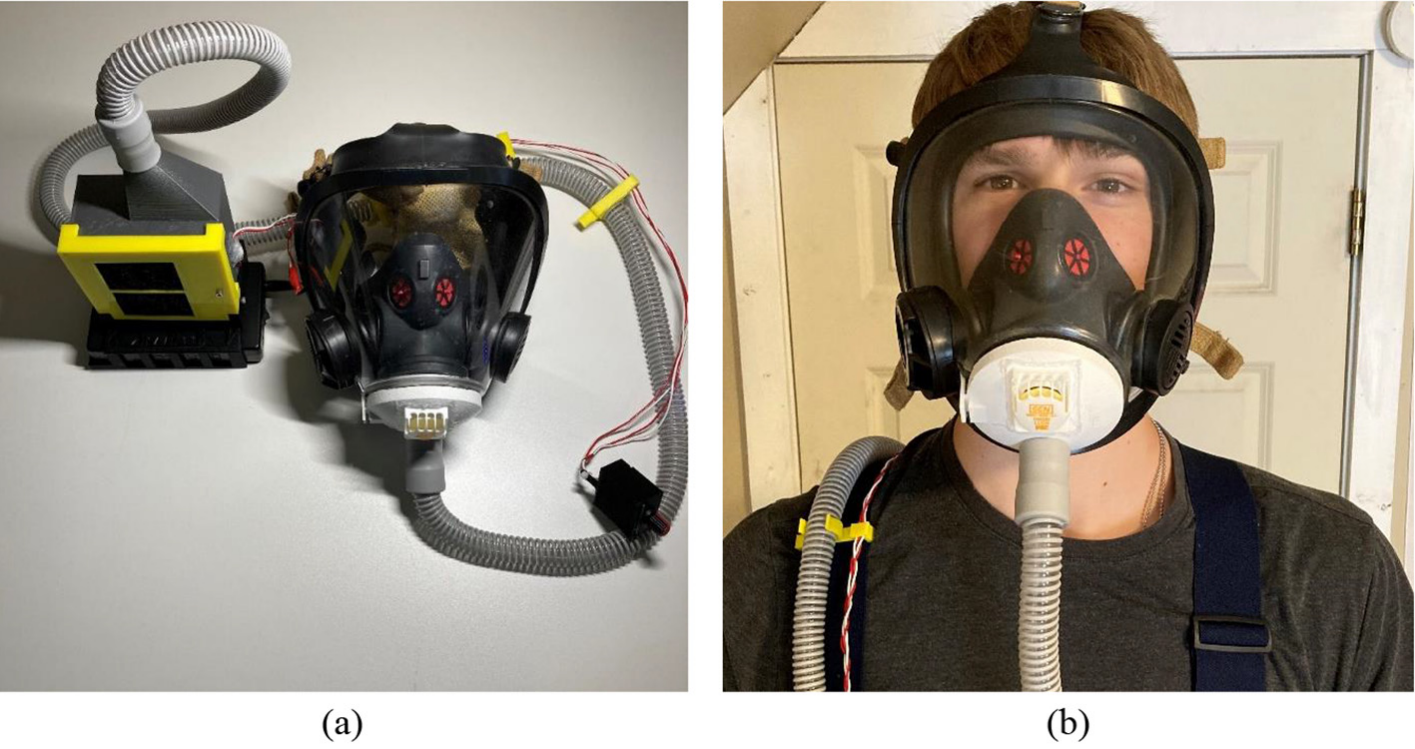
Fully assembled open source PAPR a) all components and b) in use.

The open source PAPR (Fig. 1) consists of a housing, a hose, and a respirator. The housing contains the filters, the blower, and the battery. The battery is kept well-ventilated and easily removable to allow rapid interchange of batteries in the event of a battery running out of charge. The housing is made air-tight by the use of gasket material, ensuring that all air fed to the wearer passes through the HEPA filter and optional pre-filter. The housing has a nozzle, which feeds air pushed out by the blower into a CPAP hose, which carries air to the respirator. The respirator is designed to lock into the AV3000 SCBA mask, creating an air-tight seal (once again with gasket material). With the respirator’s air inlet blocked, the mask is air-tight. The respirator has an exhalation valve to offer an easy path for exhaled air to leave the mask, keeping inhaled air fresh.

3-D printed components are designed to be easily modified to adapt to specific parts used in a particular build. Videos illustrating this process are included in the OSF repository where all project files are stored [43]. This design provides one functional conversion; however, others are possible, but caution and thought are required when interchanging parts. When selecting a different battery, ensure that it satisfies the voltage requirements of both the PWM controller and the fan, and that it has sufficient capacity to run beyond expected use times. This approach has several advantages over purchasing a proprietary PAPR:

• <$150 device replaces commercial conversion kits saving 85% or commercial PAPRs saving over 90%.
• Parametric designs allow for adaptation to other core components and equipment is wearable and custom fit specifically to fire-fighter equipment, including their suspenders
• Controllable (airflow/fan speed) and enables breathing even if fan is disconnected or battery dies.
• Battery life is 300% greater than expected usage.

## 3 Design files

### 3.1 System design files summary

**Table:** 

Design file name	File type	Open source license	Location of the file
Housing.FCStd	CAD	GNU General Public License (GPL) v3.0	https://osf.io/ydfmc/
Respirator.FCStd	CAD	GNU General Public License (GPL) v3.0	https://osf.io/ydfmc/
Suspender Clip.FCStd	CAD	GNU General Public License (GPL) v3.0	https://osf.io/ydfmc/
Controller Case.FCStd	CAD	GNU General Public License (GPL) v3.0	https://osf.io/ydfmc/

All components drawn in FreeCAD are parametric bodies. Their dimensions can be rapidly and comprehensively modified using the *param* spreadsheet contained in their respective files. Videos are provided in the Open Science Framework Videos folder detailing how to do this.

*Housing –* Contains three (3) bodies to represent the battery, fan, and filter, plus three (3) bodies to be 3-D printed - the top and bottom portions of the housing, plus a pre-filter cover, if a pre-filter is used.

*Respirator* – Contains one (1) body, the 3-D printed respirator. This part is designed to fit to the Scott Safety AV3000 SCBA mask.

*Suspender Clip –* Contains one (1) body, the suspender clip intended to manage the breathing tube and control wires.

*Controller Case* – Contains one (1) body, the controller case intended to protect the PWM controller from ESD and mechanical damage.

## 4 Bill of materials

**Table:** 

Designator/Image	Component	Number and Unit	Cost per unit - USD	Total cost - USD
**3D Printed Parts**				
	Housing Top (0.209 kg) [43]	1	$4.18	$4.18
	Housing Bottom (0.114 kg) [43]	1	$2.28	$2.28
	Pre-filter Cover (0.031 kg) [43]	1	$0.62	$0.62
	Respirator (0.054 kg) [43]	1	$1.08	$1.08
	Controller Case (0.017 kg) [43]	1	$0.34	$0.34
	Suspender Clip (0.01 kg) [43]	3	$0.20	$0.60

**Purchased Parts**				
	12v DC Centrifugal Fan97.1 mm × 95 mm × 33 mm [44]	1 ea	$84.24	$84.24
	HEPA filter, 109 mm × 72 mm × 19 mm [45]	1 ea	$3.00	$3.00
	PREFILTER OPTION 1:HEPA pre-filter, 109 mm × 72 mm [46]	0.007 sq. m	$37.44	$0.26
	PREFILTER OPTION 2:Surgical Mask [47]	1 ea	$0.46	$0.46
	Self-adhesive Foam Weather-strip 3/4″ × 3/16″ [19 mm × 4.8 mm] [48]	0.3 m	$0.69	$0.21
	M3 × 12 mm screw [49]	1 ea	$0.09	$0.09
	CPAP Hose, 19 mm ID with 22 mm ID fittings [50]	1 ea	$6.00	$6.00
	N95 Mask with 3 M Cool-Flow exhalation valve [51]	1 ea	$3.09	$3.09
	12 V DC rechargeable battery (12 V/ 6000 mAh)145 mm × 85 mm × 28 mm [52]	1 ea	$34.99	$34.99
	12 V PWM controller32mm × 35 mm × 15 mm [53]	1 ea	$4.50	$4.50
	20awg, 2wire cable/ 20awg, 4wire cableTwo 1 m lengths/ One 1 m length [54]	2 m	$0.31	$0.62

**Tools**				
	Duct Tape [55]	1 ea	$3.98	$3.98
	Electrical Tape [56]	1 ea	$5.98	$5.98
	Wire Strippers and Small Screw Driver [57]	1 ea	~$15.00	~$15.00
	Soldering Iron/Solder [58], [59]	1 ea	~$40.00	~$40.00
	Hot Glue Gun [60]	1 ea	~$13.00	~$13.00
	10 mm socket	1 ea		
	2.5 mm allen key	1 ea		

Notes on each component are included in the full bill of materials (documents\BOM.xlsx) on the OSF repository [43]. The most important of those notes are detailed here.

The fan (blower) provided in the bill of materials was selected for its ability to provide sufficient airflow at the expected static pressure of the system. Each component in the PAPR – the pre-filter, HEPA filter, housing nozzle, hose, and respirator – introduces restrictions to airflow. These restrictions result in a decrease in pressure as air traverses the component. During the design phase, the system was expected to have a pressure drop of around 50 mm H_2_O [61], meaning the fan must be able to provide a static pressure of 50 mm H_2_O for a given amount of airflow.

The required airflow for a tight-fitting PAPR (by NIOSH standards) is 4 cubic feet per minute (115 L per minute). A significant amount of literature exists to aid in understanding the process of selecting a fan/blower [62], [63], [64], [65], [66], [67], [68]. In general, axial fans (e.g. computer cooling fans) are good for large airflow rates against very low resistance (or static pressure), while centrifugal fans perform better against airflow resistance/static pressure [61], [64]. Centrifugal fans are further divided in performance characteristics by the shape and layout of their blades [65]. Ultimately, a forward-curved centrifugal fan was selected for this purpose, based on the availability of such fans made for low voltages. The data sheet for the Sanyo Denki 12 V centrifugal blower indicates that the blower can provide sufficient airflow at the expected static pressure [69]. Sanyo Denki provides the same blower in 12 V and 24 V models. The 12 V model was selected because 12 V rechargeable batteries were found to be available at lower cost than 24 V rechargeable batteries. It would be possible to design an open-source, distributed-manufacturable blower that would fit the requirements of this design, but this was outside the scope of the study and is left for future work.

3-D printed parts were designed to fit specific components, including Perry Original 2-inch suspenders and the Scott Safety AV3000 SCBA mask (may fit the AV2000 model as well (un-tested)). These parts can be modified. All 3-D printed parts were fabricated on an open source 3-D printer using approximately 0.46 kg poly-lactic acid (PLA) 3-D printer filament [70] that cost $19.99/kg. 3-D printing of these parts is discussed in detail below.

A high efficiency particulate air (HEPA) filter was selected for use in filtering the air fed into the PAPR because small HEPA filters are widely available due to their use in vacuum cleaners. According to OSHA:

[A HEPA] filter means a filter that is at least 99.97% efficient in removing monodisperse particles of 0.3 µm in diameter. The equivalent NIOSH 42 CFR 84 particulate filters are the N100, R100, and P100 filters. [71]

Where *42 CFR 84* is Title 42, Part 84 of the Code of Federal Regulations, which defines NIOSH standards for respirators, including PAPRs (in Subpart K) [72]. This means that a HEPA filter is effectively similar to the filter required by NIOSH for use in N100 non-powered APRs. This supports the selection of a HEPA filter as a low-cost filter for use in this system. **The above definition alone does not guarantee that this PAPR meets NIOSH specifications**, as there are a significant number of tests and some features required for PAPRs, and even more so for PAPRs designed for Chemical, Biological, Radiological, and Nuclear (CBRN) hazards [73].

A HEPA pre-filter or a surgical mask (pick one or the other) are optional inclusions that act as a pre-filter for the device. Pre-filters are commonly used to extend the life of HEPA filters by filtering large particulates before air reaches the HEPA filter. HEPA filters are generally replaced when they begin to significantly restrict airflow [61], [62]. Large particulates in the filter accelerate such degeneration, making a pre-filter useful for optimizing the life of the more expensive HEPA filter. The drawback of pre-filters is that they add resistance to airflow, which can bring airflow below NIOSH requirements. Testing has shown that the surgical mask brings airflow below NIOSH specifications, but the HEPA pre-filter does not. However, the HEPA pre-filter may be less available and is a large initial investment (purchased in 0.5 square meter sheets for around $17) [46], which is why the surgical mask was explored as a secondary option. Note that pre-filters are commonly changed out, and the sheet of HEPA prefilter can be used to 68 total pre-filters, so the cost is made up over the course of use (particularly if being used by multiple systems as in a fire department). Cost and airflow requirements must be weighed to determine whether airflow or filter-life are desired.

A CPAP hose is used for several reasons. First, CPAP is a well-established technology, making hoses readily available. The fittings on CPAP hoses are a standard size, and the hose included in the BOM has rubber fitting that creates a tight seal on the nozzle it connects to. In addition, CPAP hoses have a smooth inner wall, minimizing airflow resistance due to friction [74].

The Scott Safety AV3000 mask is a tight-fitting mask, making the PAPR a tight-fitting PAPR [72]. This means that an outlet for exhaled air should be included on the respirator. An exhalation valve such as the 3 M Cool-Flow valve is a simple valve that acts as a diode, allowing air to flow out, but not in. The 3 M Cool-Flow valve was selected because it is used in NIOSH-certified N95 respirators, suggesting it performs well. An open-source valve could be developed in future work, but that was beyond the scope of this design, and additional testing would be required to validate such a valve. An exhalation valve allows air to exit the mask with minimal resistance, minimizing the risk of CO_2_ rebreathing [75] and allowing the fan to perform well. One downside of an exhalation valve is that the breather’s exhaled air enters the environment unfiltered [76]. In the case that the wearer of this PAPR is or could be contagious, such behavior is not desirable. The exhalation valve may be replaced with another filter, such as a cutout from an N95 mask. Note that this will decrease airflow from the PAPR, likely to levels below NIOSH standards. This has not been tested and is left for future work.

## 5 Build instructions

### 5.1 3-D printing

The components were 3-D printed on an Athena II Delta printer [77], [78] and a Lulzbot TAZ 6 [79], each of which is a self-replicating rapid prototyper (RepRap)-class open source 3-D printer [80], [81], [82]. All 3-D printed components were printed from polylactic acid (PLA), which has an average tensile strength of 56.6 MPa and an elastic modulus of 3368 MPa [83]. The STL files for the parts printed on the Athena II were sliced using open source Ultimaker Cura 4.6.1 [84] using the print settings in Table 1. The STL files for the parts printed on the TAZ 6 were sliced using open source Cura Lulzbot Edition 3.6.20 [85] using settings in Table 1. Details such as bed adhesion and retraction should be selected to meet the needs of the printer in use (what was used here is detailed in Table 1).

**Table 1:** 3D-Printer Settings.

Setting	Athena II	TAZ 6
Print Temperature	210 °C	205 °C
Layer Height	0.2 mm	0.25 mm
Wall Thickness	2 mm	2 mm
Top/Bottom Thickness	0.8 mm	2 mm
Infill	10% Cubic	20% Cubic
Infill/Inner Wall Speed	70 mm/s	60 mm/s
Top/Bottom/Outer Wall Speed	35 mm/s	40 mm/s
Bed Adhesion	Raft	Skirt
Supports	Varies by part, noted below	

There are many floating surfaces in these designs, however, little support is actually needed due to the design of the parts. Note that some cleanup will be required, but the material savings allowed by the lack of support is significant. Such floating designs tend to find greater success at mid-to-low print speeds. Each part is shown with necessary support material below.

**Fig. 2:**
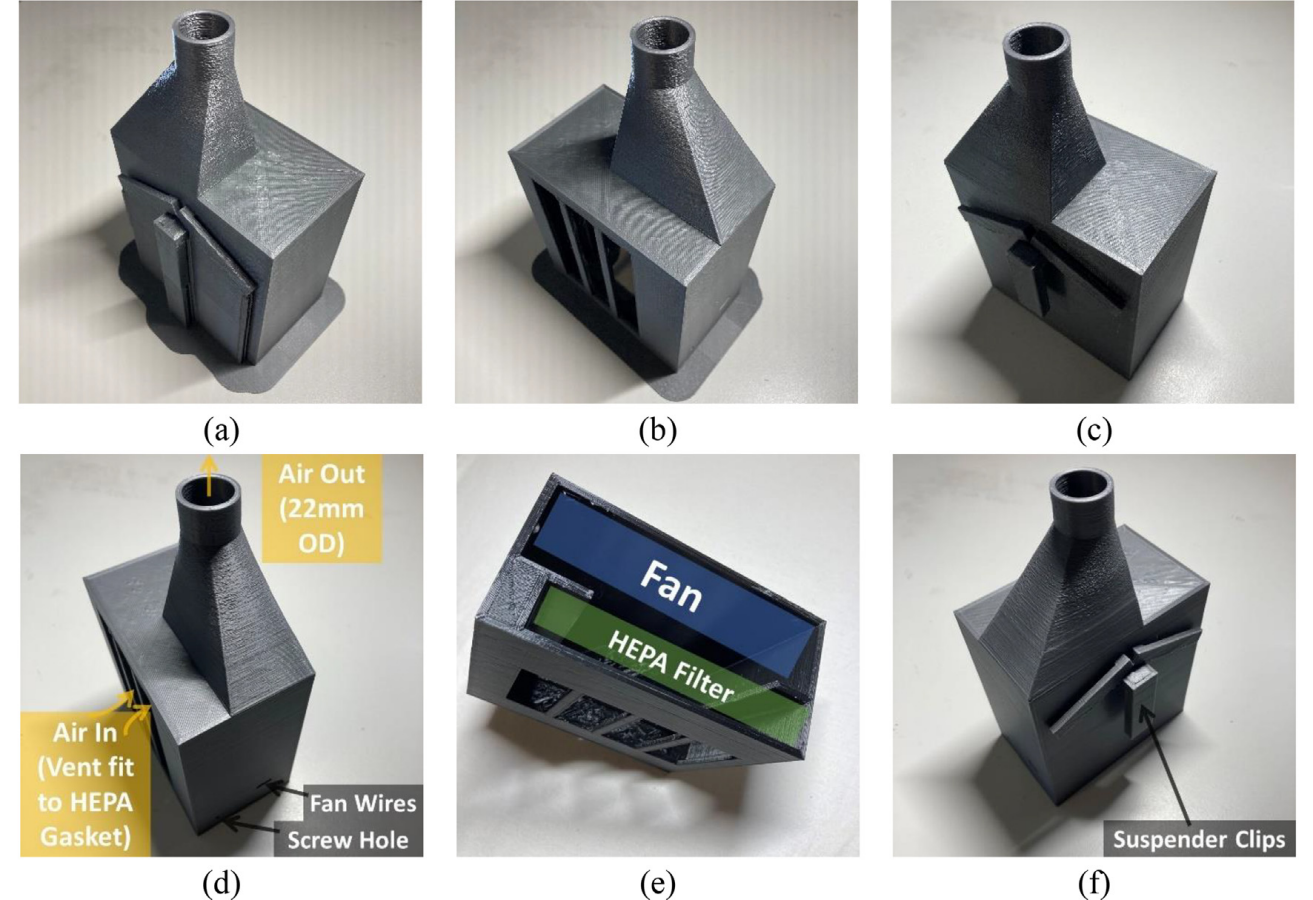
Housing Top (a, b) Before cleanup; (c) After cleanup (d, e, f) Design details.

The **housing top** is shown in Fig. 2. This should be printed with the nozzle on the top, and the rectangular opening on the build plate. The suspender clips on the back are the only features that require support. This part requires **build plate supports on the outside only** (for the suspender clips only).

**Fig. 3:**
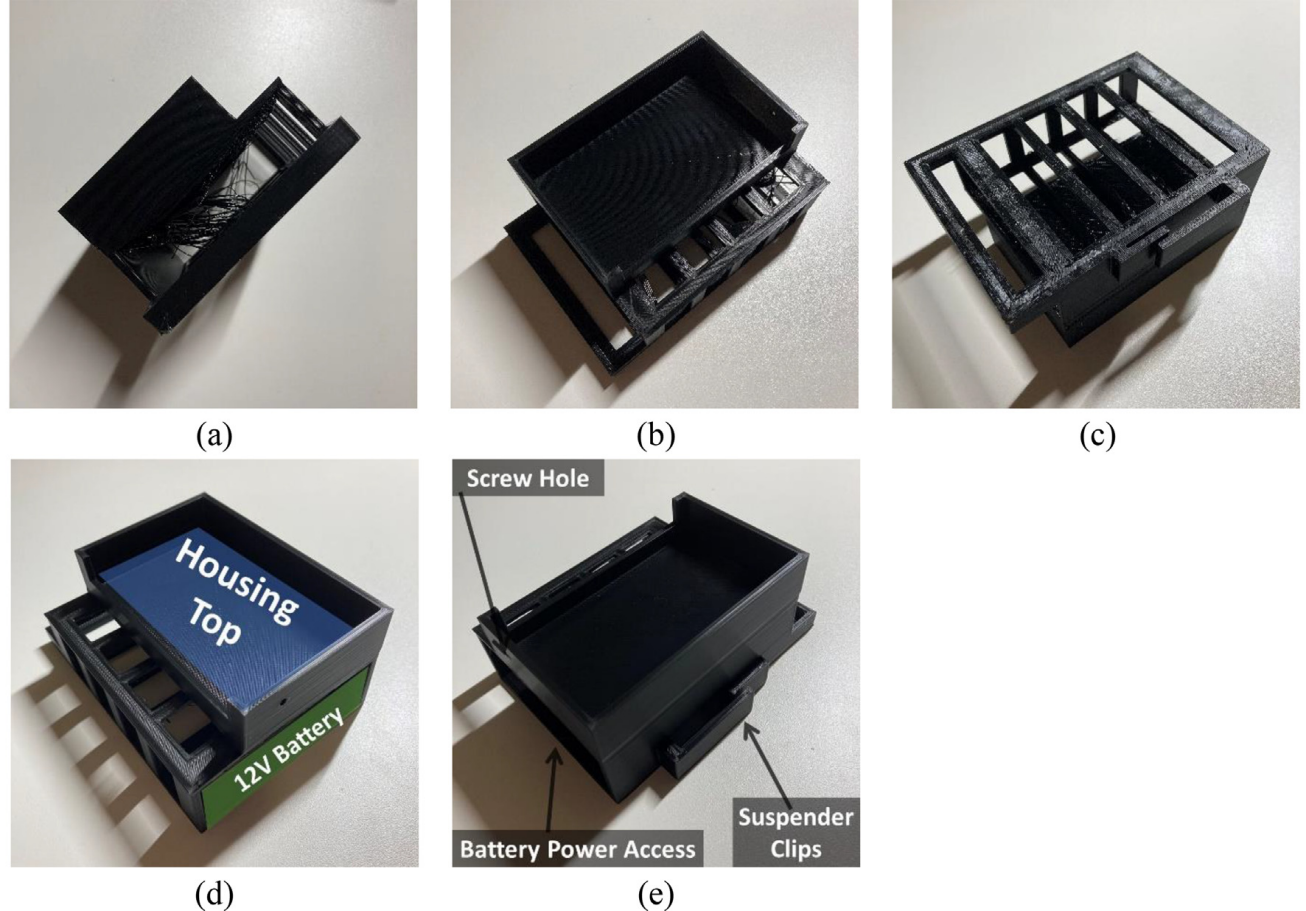
Housing Bottom (a, b) Before cleanup; (c) After cleanup (d, e) Design details.

The **housing bottom** is shown in Fig. 3. This should be printed with the large rectangular opening upward and the long thin strips of the battery cage on the build-plate. The build is self-supporting when printed at low/medium speed – there will be some cleanup necessary, but this part requires **no supports**. When cleaning the part, it is okay to leave some strands inside the battery cage – this will help hold the battery in place.

**Fig. 4:**
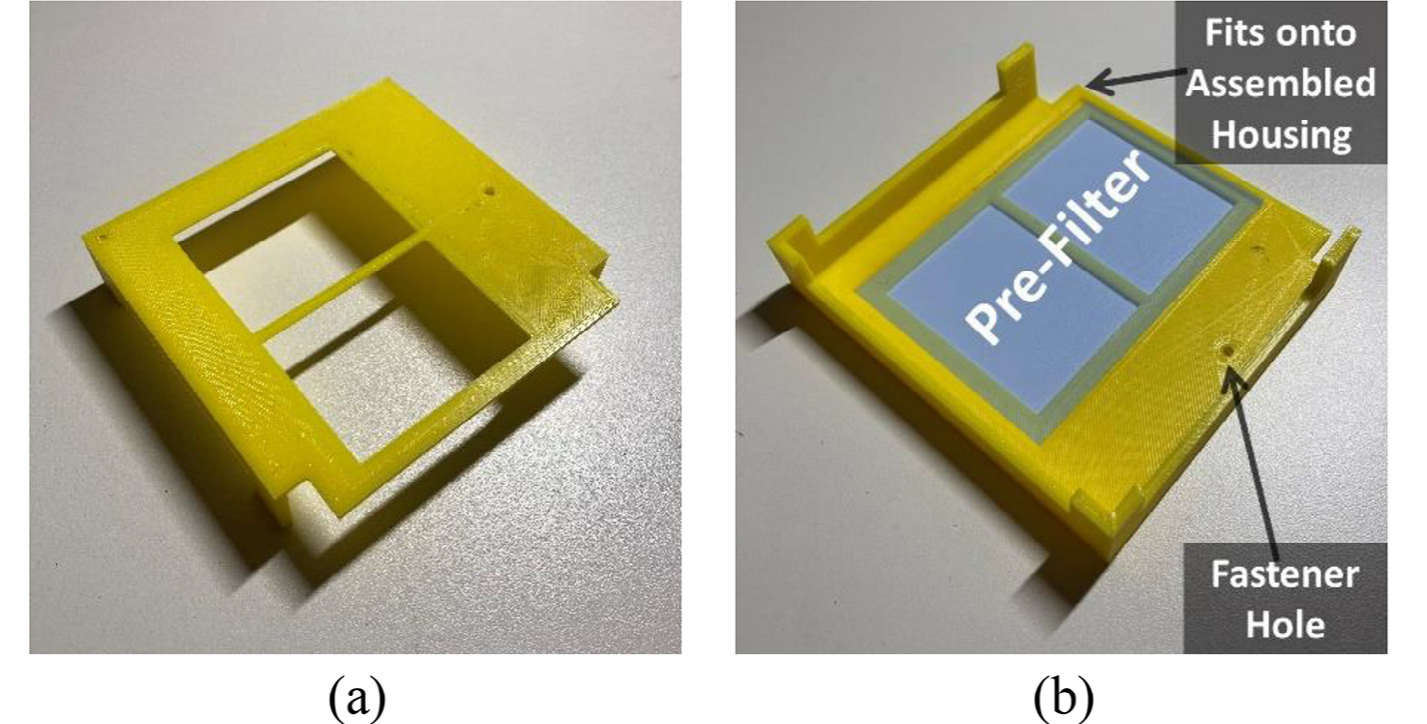
Pre-filter cover.

The **pre-filter Cover** is shown in Fig. 4. This should be printed with the flat surface on the build plate, requiring **no supports**.

**Fig. 5:**
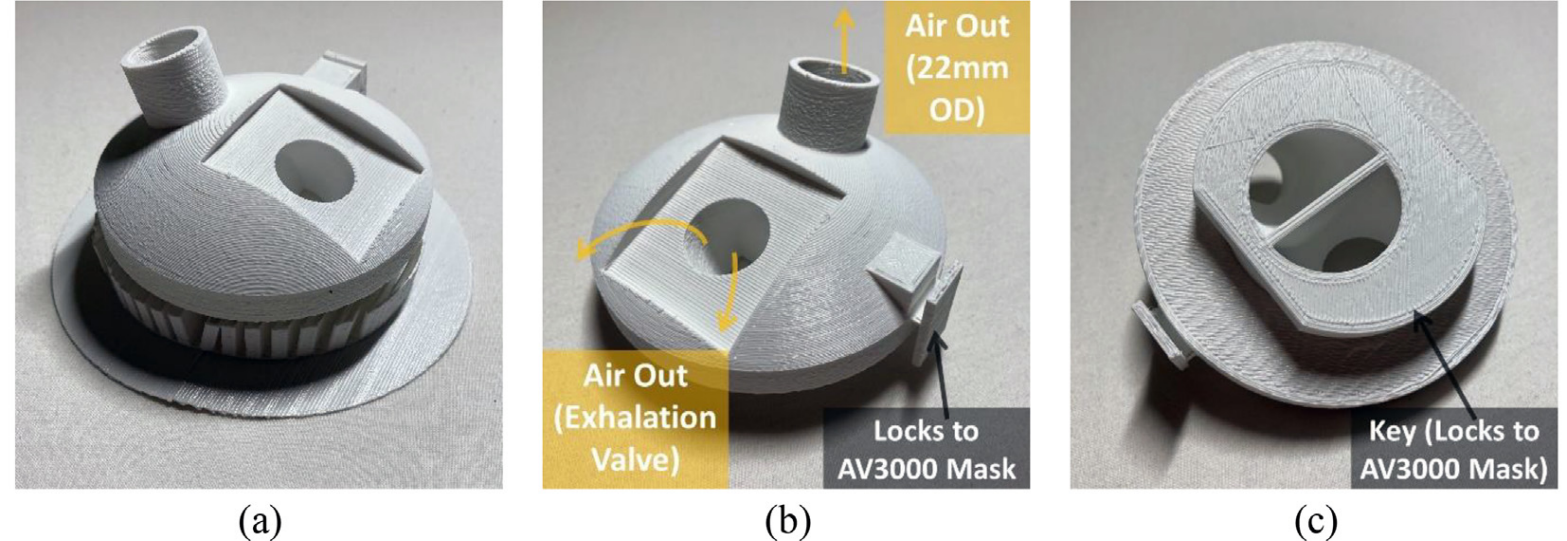
Respirator (a) Before cleanup; (b, c) After cleanup with design details.

The **respirator** is shown in Fig. 5. This should be printed with the flat surface on the build plate and the domed surface upward. This part requires **build plate supports only** – this will support the circular surface that spans wider than the locking mechanism.

**Fig. 6:**
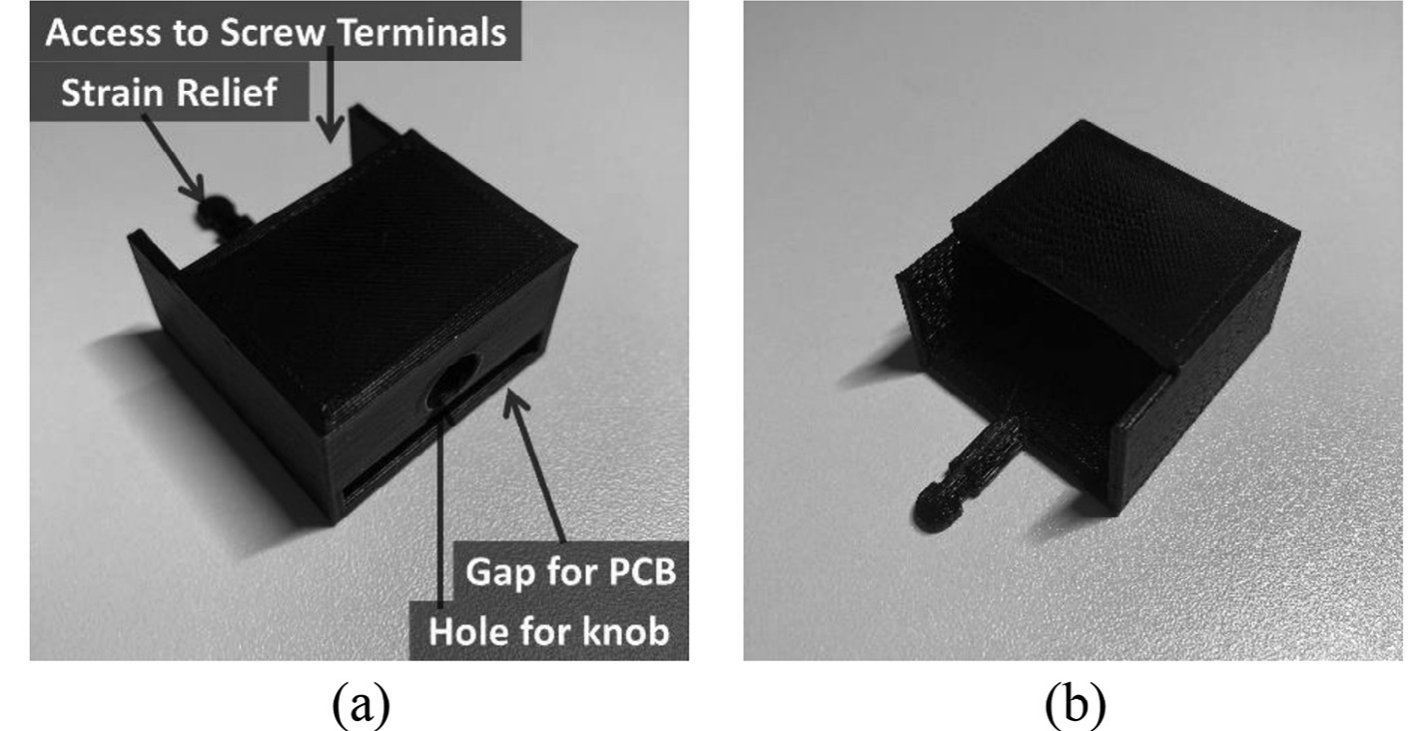
Controller Case (a, b) with design details.

The **controller case** is shown in Fig. 6. This should be printed with the largest surface on the build plate. This part requires **no supports**.

**Fig. 7:**
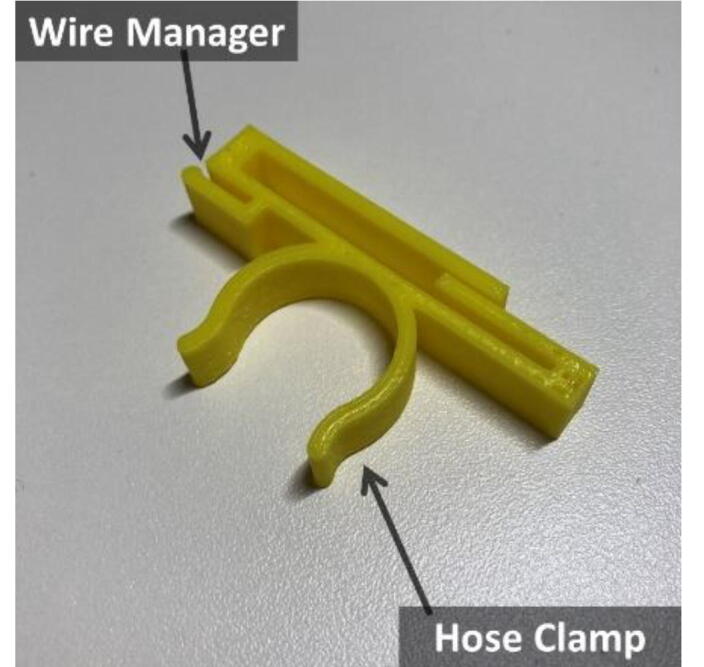
Suspender Clips (a, b) with design details.

The **suspender clip** is shown in Fig. 7. This should be printed with its side on the build plate, allowing the part to be printed with **no supports**.

While parts are printing, other prep-work can be completed.

### 5.2 Part preparation

**Fig. 8:**
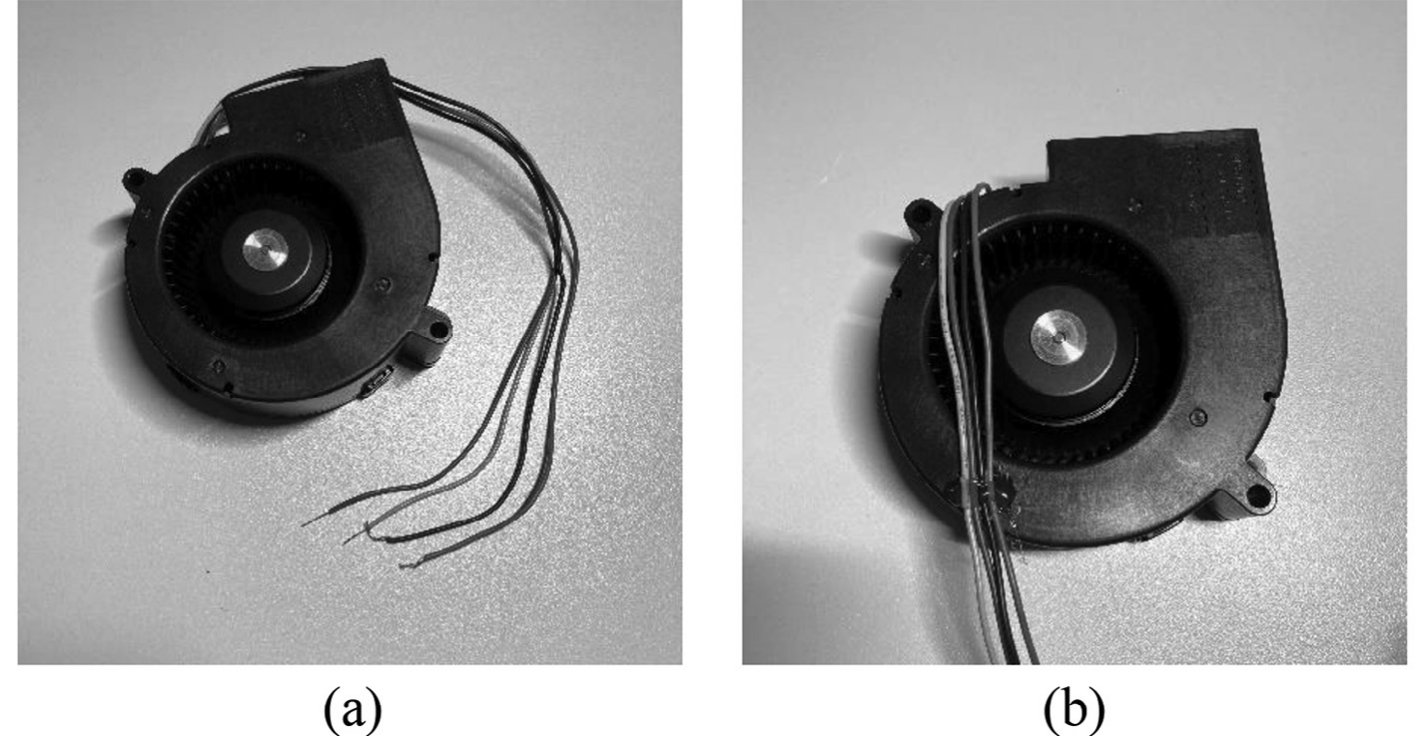
(a) Cut and strip fan wires. (b) glue the fan wires in place.

To prepare the fan for use, remove it from its packaging. If the wires are terminated in any way, cut and strip the wire ends, keeping the maximum length of wire connected to the fan. This extra length will allow the fan to be removed from the housing without breaking wire joints created later. Strip the wires back about 10 mm (Fig. 8a). These will be connected to a longer run of wire with a butt joint.

The fan will slide into the housing, and the wires will exit the housing below the out port of the fan. Depending on the location of the wires on the fan, it may be necessary to fasten them to the body of the fan to allow the fan to fit into the housing. For this fan, the wires were pulled straight downward and hot glued in place (Fig. 8b). This takes advantage of the gap in the housing between the fan and the HEPA filter. The need for such fastening can be assessed once the housing top is printed.

Once the fan is prepared, place the fan back in its packaging for safe keeping – it is important to keep particulates out of the fan blades, as they could damage the blades when the fan is running.

**Fig. 9:**
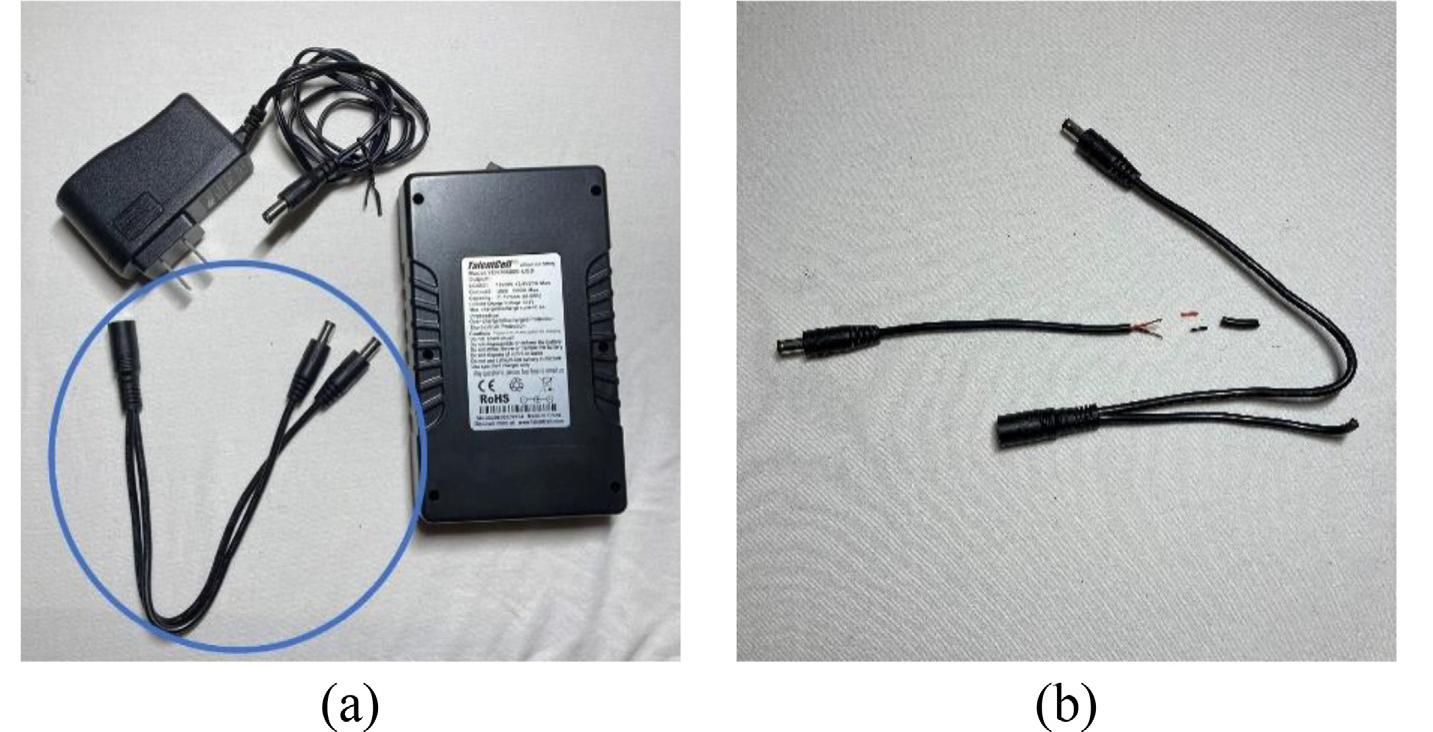
12 V DC connector (a) provided with the battery; (b) cut off and prepare a male connector. The remaining connectors are not used.

The 12 V battery should ship with a 12 V DC power splitter as shown in Fig. 9. This will be used to scavenge a 12 V connector to link the fan and the battery. If the battery does not include such a connector, then an extra connector must be purchased or cut off an *extra* power supply – while you could use the connector from the power supply provided with the battery, **this is not recommended**, as it would make recharging the battery a challenge. Take the 12 V DC power splitter provided with the battery and cut off one of the male connectors, then strip back the wires as done for the fan.

**Fig. 10:**
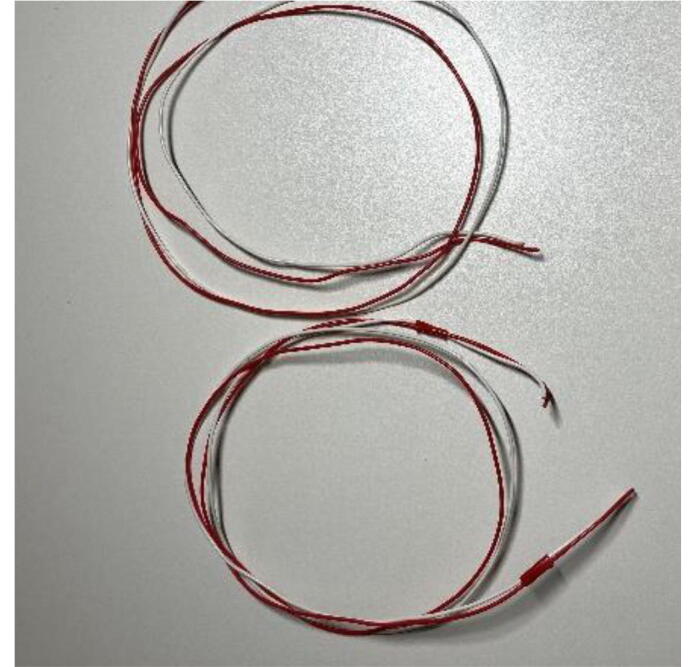
Controller cables; cut to length, stripped, and marked.

Cut two 1-meter lengths of 2-wire 20 AWG cable (or one 1-meter length of 4-wire 20 AWG cable) (Fig. 10). This cable will run from the housing, which sits between the shoulder-blades of the wearer, up over the shoulder of the wearer to provide access to the blower speed controller. Use discretion in selecting the lengths, but it is recommended that the runs are kept longer to begin with, allowing them to be shortened if necessary.

After cutting to length, strip back the wires. One end will be fit into screw terminals and should be stripped to around 5 mm in length – if they are stripped too far back, there is an increased risk of shorting out wires at the screw terminals on the controller. The other end will be joined with the fan or 12 V connector with a butt joint and should be stripped to 5–10 mm in length. Wrap one of the wire pairs in red or black electrical tape on either end of the cable to indicate that it connects to the power supply.

**Fig. 11:**
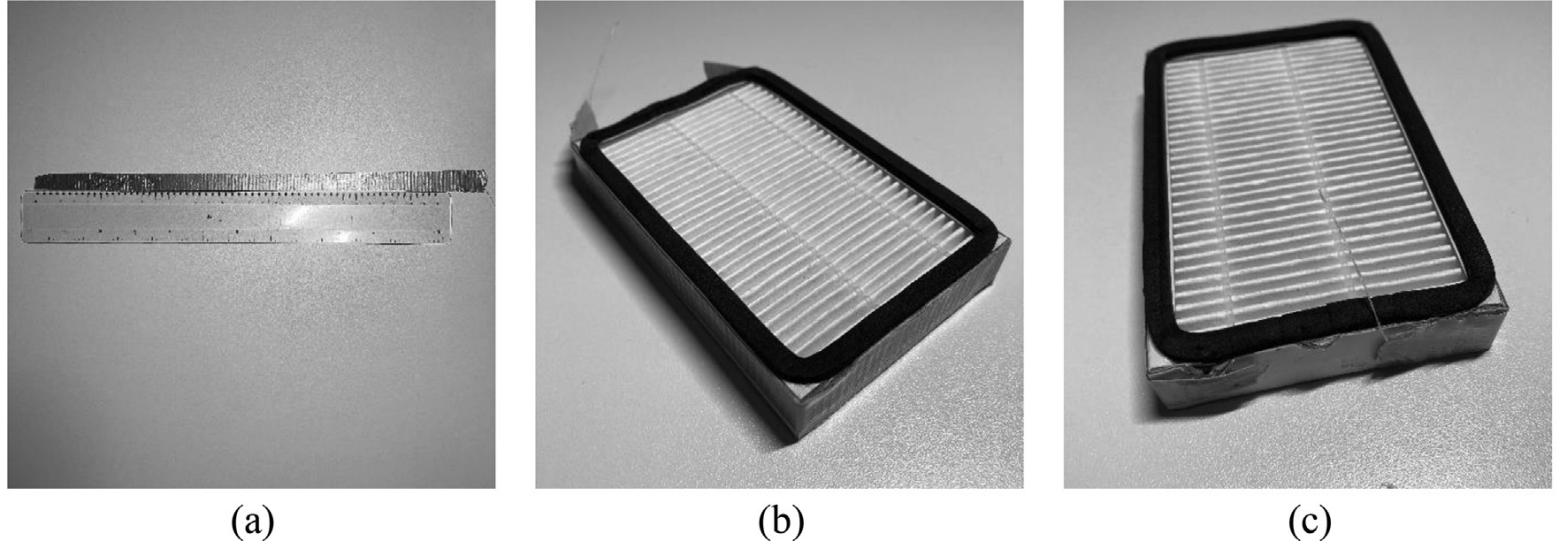
(a) Cut duct tape to length (b, c) apply to HEPA filter.

The HEPA filter will be interference fit into the housing [86]. Due to the need for the housing to be air-tight, the fragility of the filter, and built-in adaptability to handle other filter and fan sizes (through parametric design) there are no grips built in to grab the filter when removing it. Instead, a high tensile-strength tape, such as duct tape, is used as a handle to pull the filter out for replacement. Cut a 10 mm × 320 mm strip of duct tape, and apply it to the HEPA filter, leaving the two ends available as tabs at the face of the filter which will be exposed when installed in the housing (Fig. 11).

**Fig. 12:**
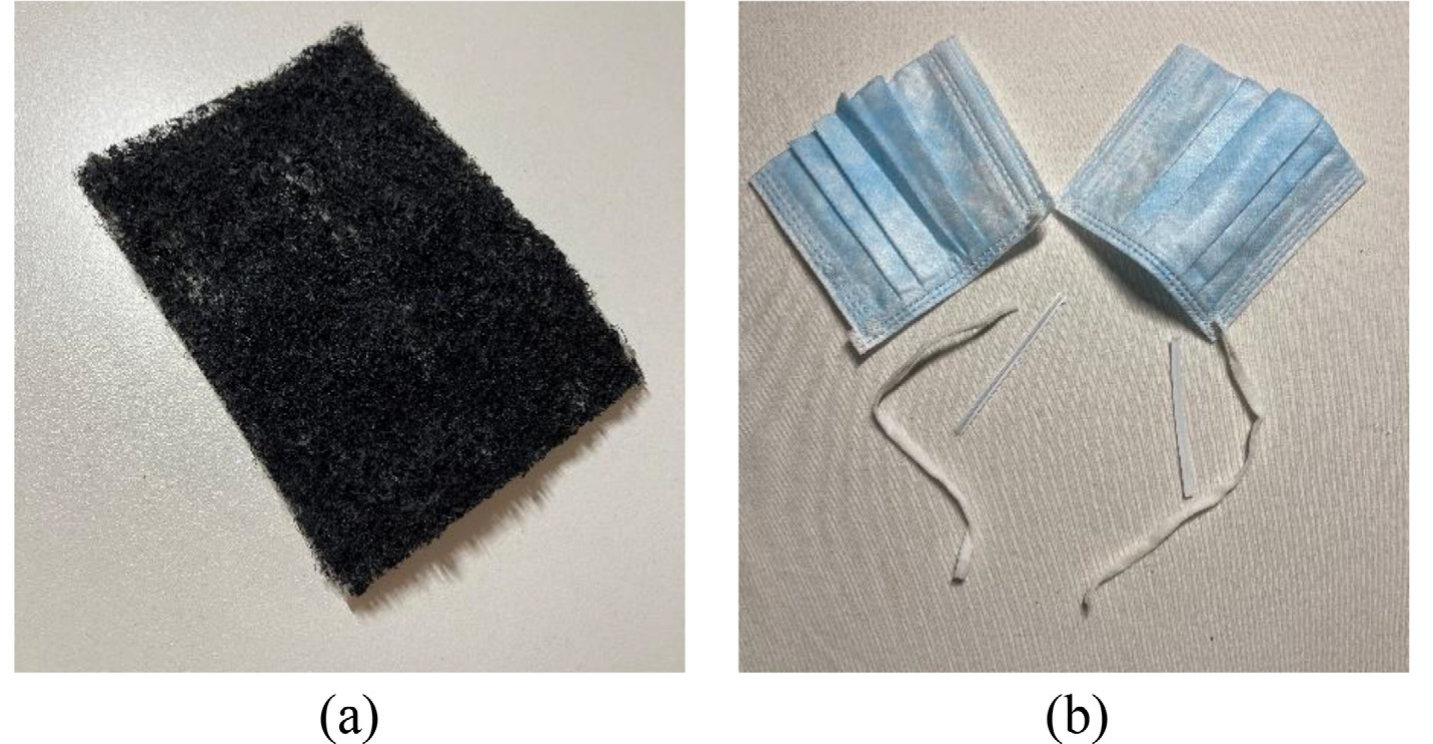
Pre-filter (a) HEPA pre-filter; (b) surgical mask.

A pre-filter may be included to extend the life of the HEPA filter. The pre-filter should be a HEPA pre-filter to minimize the impediment of airflow. In the event that HEPA pre-filters are not available, a surgical mask may be used instead. Note that the surgical mask will reduce airflow below NIOSH standards. The pre-filter should be cut to the dimensions of the pre-filter cover (95 mm × 68 mm) (Fig. 12a). When cutting the HEPA pre-filter, be sure to consider the orientation of the cutout which will maximize the number of pre-filters which can be cut from the sheet. If instead using a surgical mask, simply cut it in half and remove any wire reinforcement (Fig. 12b).

**Fig. 13:**
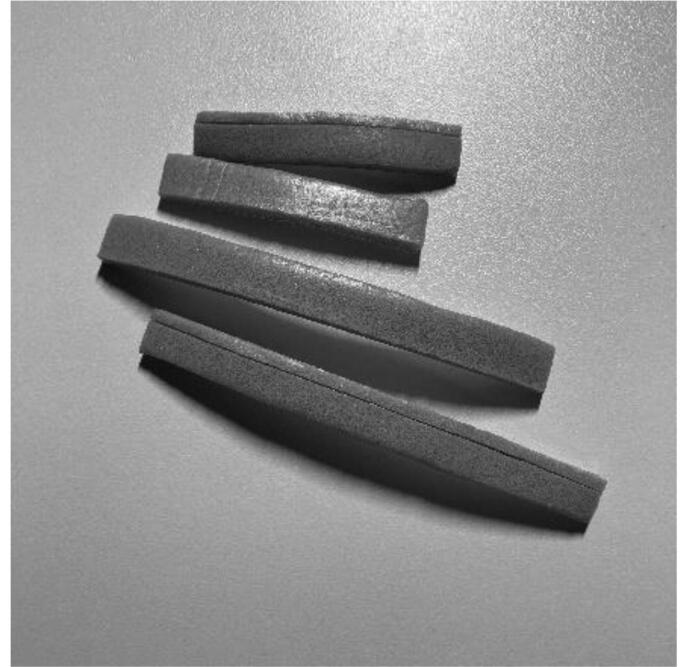
Weather-seal gasket, cut in half lengthwise.

Cut three lengths of weather stripping: 60 mm, 105 mm, and 115 mm. These will be used as gasket material to achieve an airtight seal. The 115 mm length will be used on the respirator and the other two will be used to line the seam between the housing top and bottom. Cut each of these strips in half lengthwise, resulting in two strips of each length, about 2.5 mm wide (Fig. 13).

**Fig. 14:**
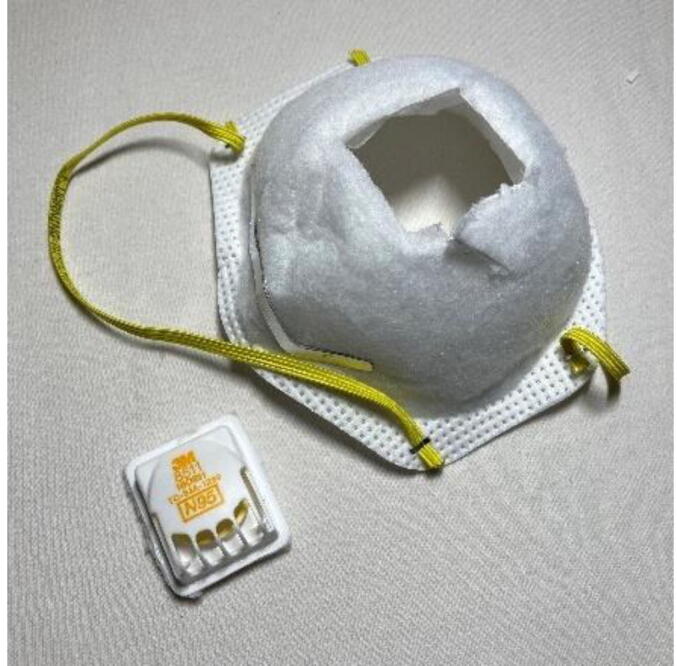
3 M Cool-Flow Exhalation Valve removed from mask.

A 3 M Cool-Flow exhalation valve from a 3 M N95 mask [87] is used on the respirator for venting exhaled air. Take the N95 mask and cut out the 3 M Cool-Flow from the mask, keeping only the plastic valve (any excess material could prevent the valve from fitting onto the respirator (Fig. 14). In the case that exhaled air should be filtered, cut a square of N95 material to the same dimensions as the exhalation valve (37 mm × 43 mm).

### 5.3 Assembly

After printing and cleaning all 3-D printed components and completing all other part preparation, assembly can begin.

The respirator can be completed quickly. Its assembly requires the 115 mm strips of gasket material, the 3 M Cool-Flow exhalation valve, hot glue, and masking tape.

**Fig. 15:**
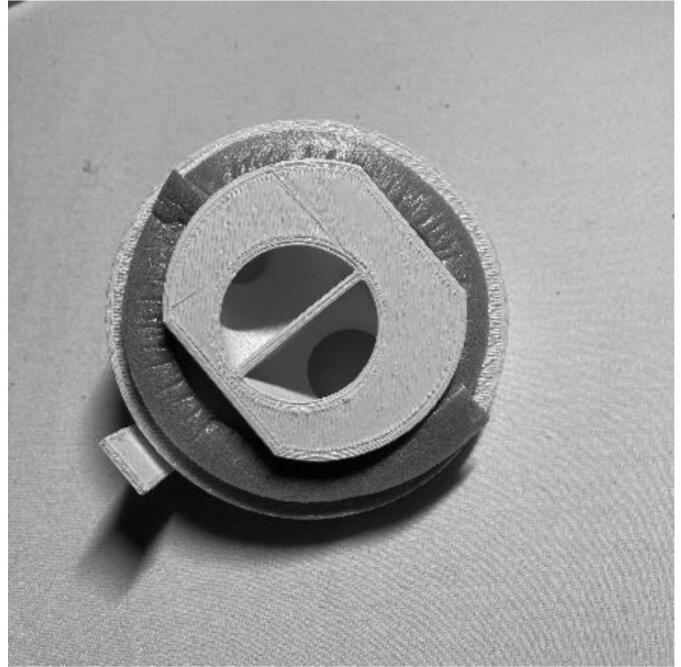
Gasket applied to the respirator.

First, take the 115 mm strip of gasket material and fit it onto the flat, overhanging surface of the respirator as shown in Fig. 15. Trace the arc of the key (a 60 mm circle) rather than keeping tight to the key where it flattens off. This is important because the AV-3000 facepiece has two circular ridges on the front of the mask to create a seal with the gasket – these must come in contact for a good seal. This can be checked by fitting the respirator onto the mask.

**Fig. 16:**
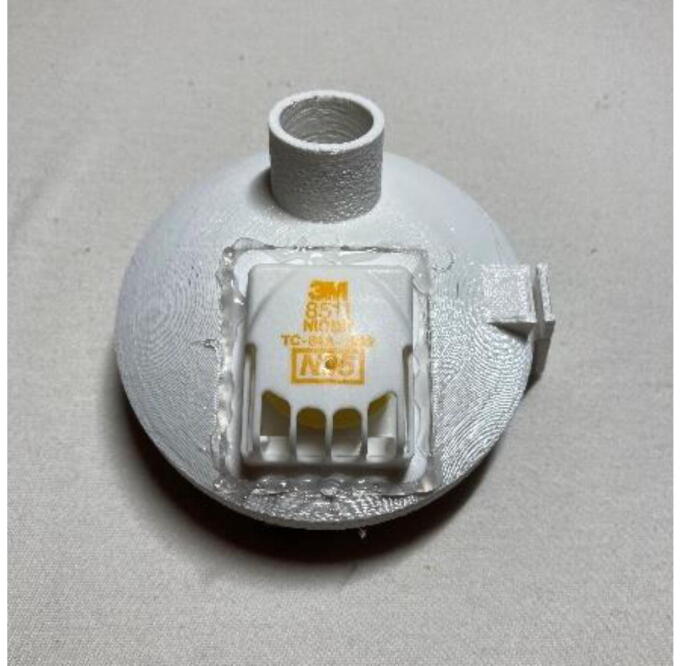
Glued 3 M Cool-Flow exhalation valve in place.

Next, use hot glue to fix the exhalation valve inside the rectangular pocket on the domed surface of the respirator. Apply glue to the entire perimeter to create an air-tight seal as shown in Fig. 16. Other adhesives may be used, but they must create a seal around the perimeter of the valve, allowing air to flow only through the valve, and not in through the cut edges.

**Fig. 17:**
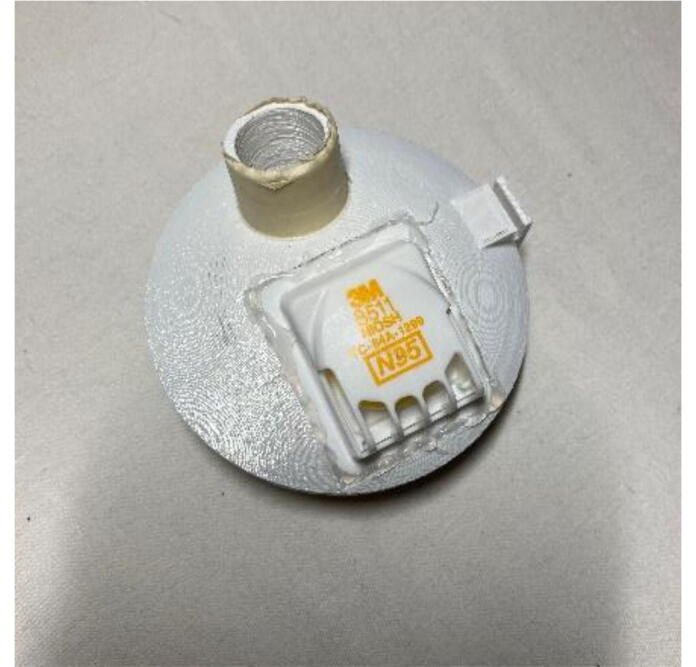
Apply tape to the nozzle to provide and air-tight fit to the hose.

Finally, fit the hose to the respirator nozzle. For a CPAP hose with a rubber connector, the fitting should be snug and airtight. For hoses with hard plastic connectors, the nozzle is expected to print undersized. This leaves room to add tape to realize a snug fit, if necessary (Fig. 17). Masking tape was used here and showed no signs of leaking; plumber’s tape or duct tape could also be used to ensure an air-tight seal.

Next, assemble the housing top. This assembly will involve the 3-D printed housing top, the 12 V centrifugal fan, the 12 V connector, the unmarked (no electrical tape) meter of 2-wire cable, solder/soldering iron, the HEPA filter, and hot glue.

First, keeping the fan *outside* the housing, feed the ends of the fan wires through the rectangular wire hole on the side of the housing. Feed approximately 25 mm of wire through. This should allow the fan to sit comfortably outside the housing.

**Fig. 18:**
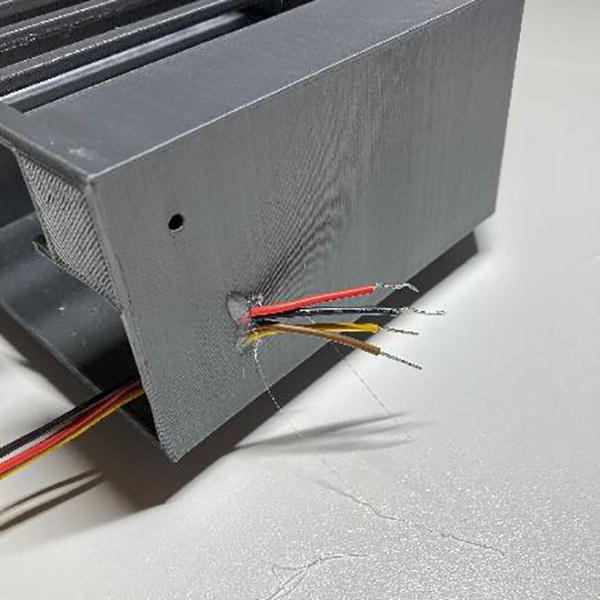
Glued wires in place in case.

Use hot glue to fix the wires in place as shown in Fig. 18. Apply a judicious amount to ensure an air-tight seal is formed around the wires. Keep in mind while applying the glue that the fan must slide into the housing top, past the wires and any glue inside.

Next, slide the fan into the housing, adjusting the slack on the fan wires as necessary to keep them loose and out of the way.

**Fig. 19:**
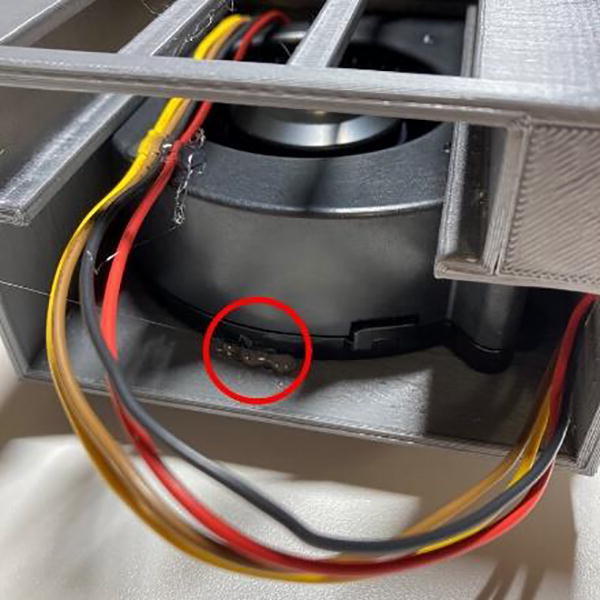
Glued fan in place (if necessary).

Apply a small amount of glue along the bottom of the fan (one or two spots is sufficient) to hold the fan in place (Fig. 19). When applying the glue, be sure that it does not bulge out below the bottom ridge of the housing, as this will create gaps for unfiltered air to enter the housing during operation. If necessary, use a flat object such as a knife or ruler to skim down hot glue while it is still viscous. Note that this is not relevant if using the parts specified in the BOM because the HEPA filter is taller than the fan.

**Fig. 20:**
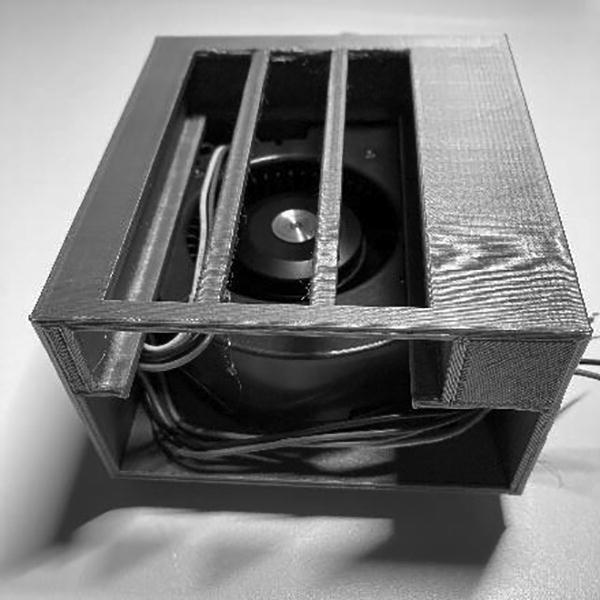
Properly run wire management.

Once the fan is in, twist and tuck the excess wire into the housing (Fig. 20).

Next, attach the unmarked 20 AWG wire to the fan wires and the marked 20 AWG wire to the 12 V connector, being careful to note the polarity of the wires. If using a single 4-wire cable, be sure to connect the wires so that the 12 V connector is on the same end as (next to) the fan wires, leaving four bare wires on the other end of the cable.

Wire joining can be accomplished in a variety of ways, including crimp-on butt connectors, wire nuts, and solder. Here, solder was used because it is the most permanent and sturdy connection. This is done prior to inserting the HEPA filter to avoid risk of damaging the filter with heat or flux.

**Fig. 21:**
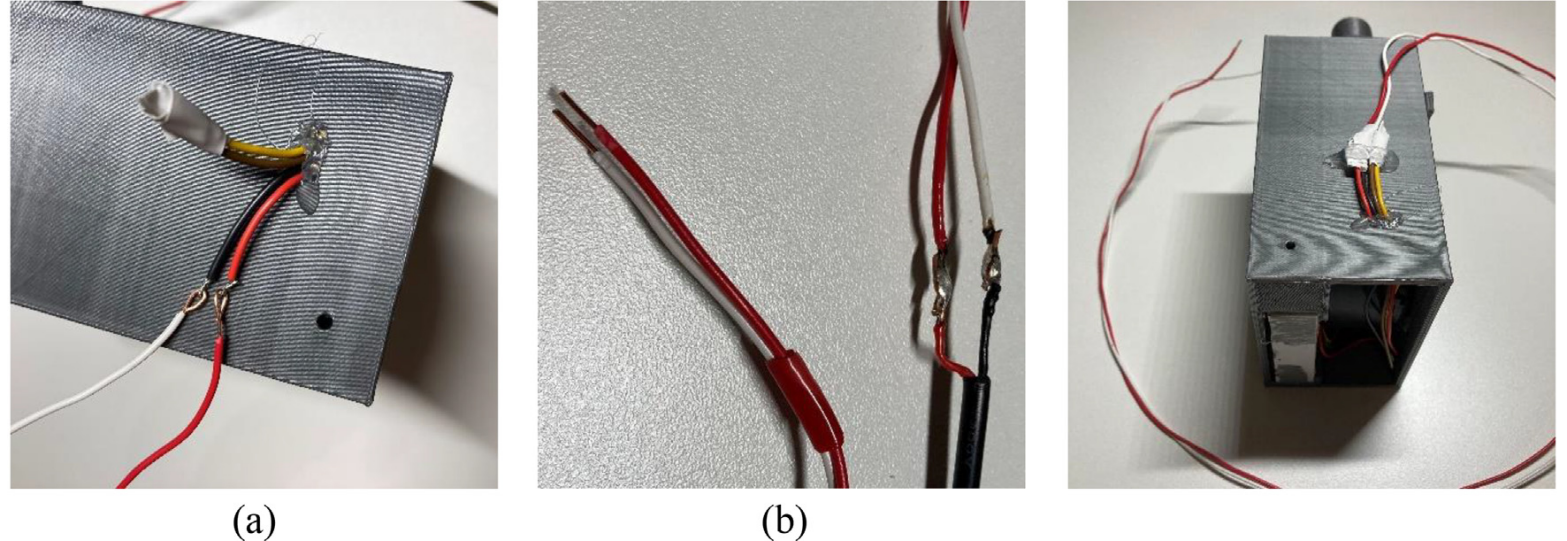
Wire joining (a) form hooks; (b) solder; (c) wrap and glue.

Strip the wires back to around 10 mm in length, taping off any unused signal wires from the fan (yellow and brown for this fan). Electrical tape or heat-shrink tubing will be used to cover the joint after it is complete. If using heat shrink, slide it over the wire and far from the wire end (to ensure it is not heated by soldering). Form hooks with the wire ends. Hook the wires together and clamp down the loops, securing the wire ends together (Fig. 21a). Solder the wires together, being sure that the solder closes the loops and secures the hooks together (Fig. 21b). Apply the heat shrink tubing or wrap the joint in electrical tape, covering all exposed conductor to ensure that the wires cannot short out, then use hot glue to fix the joint onto the housing with the wires running up toward the nozzle. This will help prevent the fan wires from being strained and will protect the solder joint. Lay a dollop of glue on the side of the housing top directly above the wire slot, then set the wires into the glue. Apply more glue over the top, then hold in place until the glue sets (Fig. 21c).

**Fig. 22:**
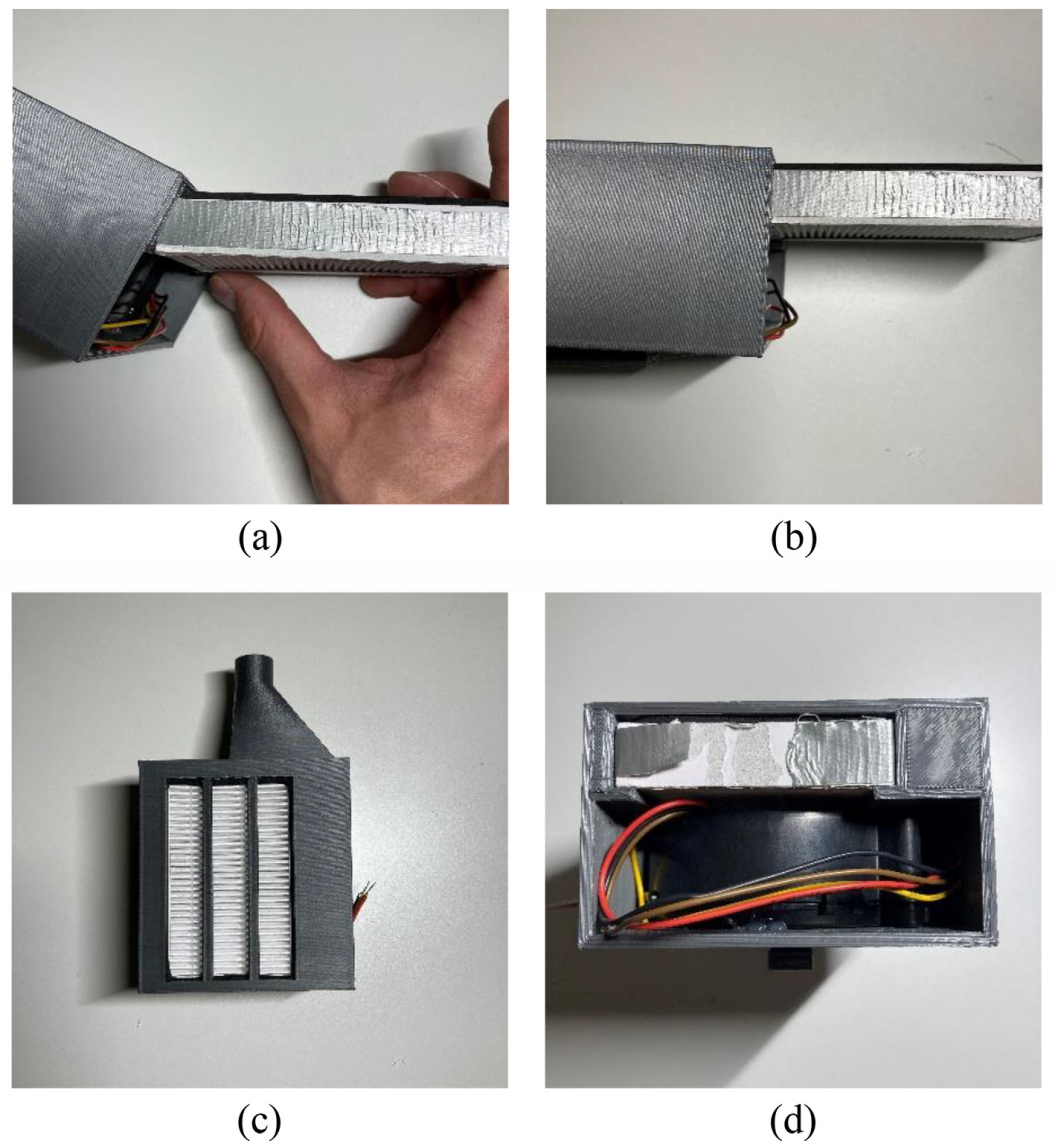
HEPA filter installation (a) engage gasket; (b) compress gasket; (c, d) slide into place.

Next, insert the HEPA filter (Fig. 22), being sure to keep the duct tape tabs (to be used for removal) exposed as shown in Fig. 22d. The filter comes with a gasket to ensure that air cannot circumvent the filter. The housing is designed intentionally to have a compressive fit on the filter to make use of that gasket. For the gasket to be effective, it must be on the side facing the exterior of the housing. To fit the HEPA filter inside the housing, tilt the filter and engage the gasket with the inner surface of the housing (Fig. 22a). Tilt the filter down, flattening it out, and press upward on the back of the filter, compressing the gasket and fitting the profile of the filter into the housing (Fig. 22b). Slide the filter into the housing, tucking the gasket under edges and being careful not to dislodge or scrape the gasket from the filter body. Align the filter with the window on the side of the housing, ensuring the gasket is in contact with the housing all around the window (Fig. 22c).

This completes the assembly of the housing top. Next, the controller can be connected to the fan. This will require the housing top (with the meter run of wires already attached), the marked length of cable with the 12 V connector attached, the 12 V PWM controller, the 3-D printed controller case, a zip tie, a 10 mm socket or open-ended wrench, and a small screwdriver (flathead or Philips).

**Fig. 23:**
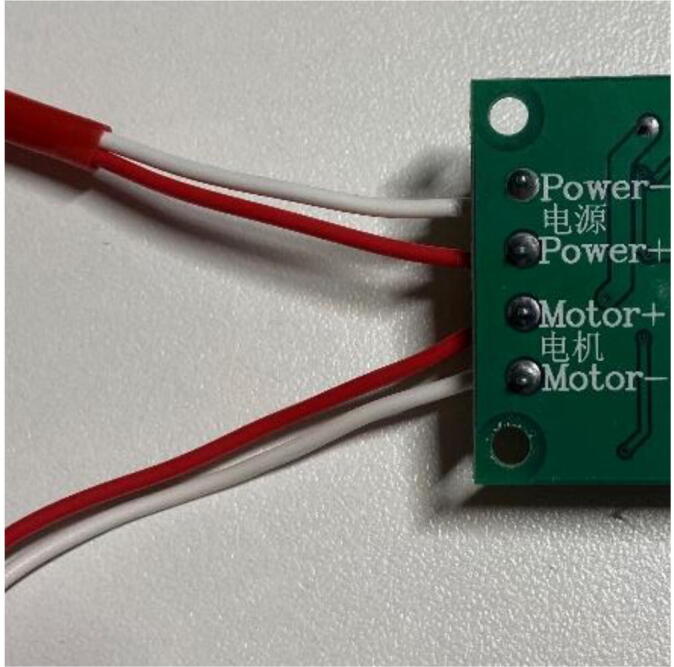
Connect the wires to the controller.

If not already loose, use a small screwdriver to loosen the 4 screws on the PWM controller’s screw terminals. Insert the four bare wires from the two 1 m lengths of cable into their respective slots, tightening the screw terminals to secure the wires in place as shown in Fig. 23. **Be very careful of polarity – the controller will sustain damage if provided with inverted power** [53]**. Be similarly careful that no strands of wire enter other terminals.** Ensure the wires are securely attached by giving them a sturdy tug.

**Fig. 24:**
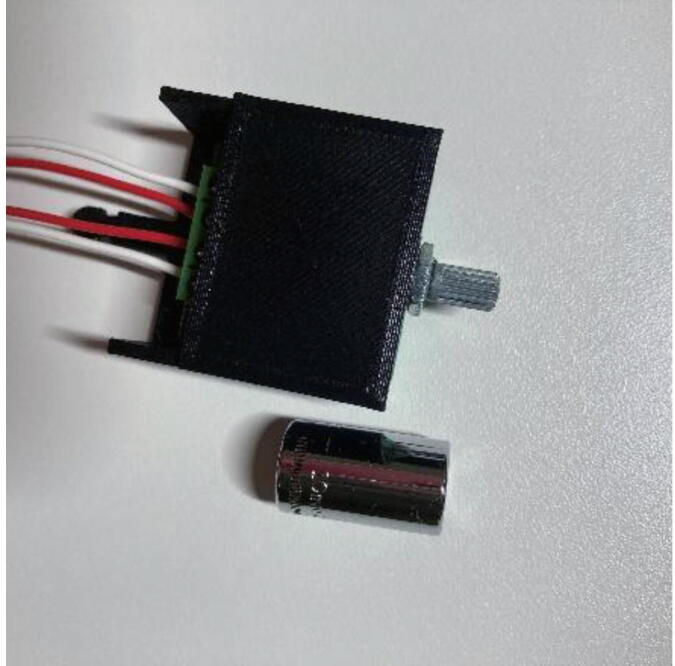
Fix the PCB into the controller case.

With the wires secured in place, fix the controller into the controller case using the washer and nut provided with the controller. Due to the layout of the board, the PCB must fit into the slot along the base of the controller case for the threads on the nut to engage. Tighten the nut with a 10 mm socket (Fig. 24).

**Fig. 25:**
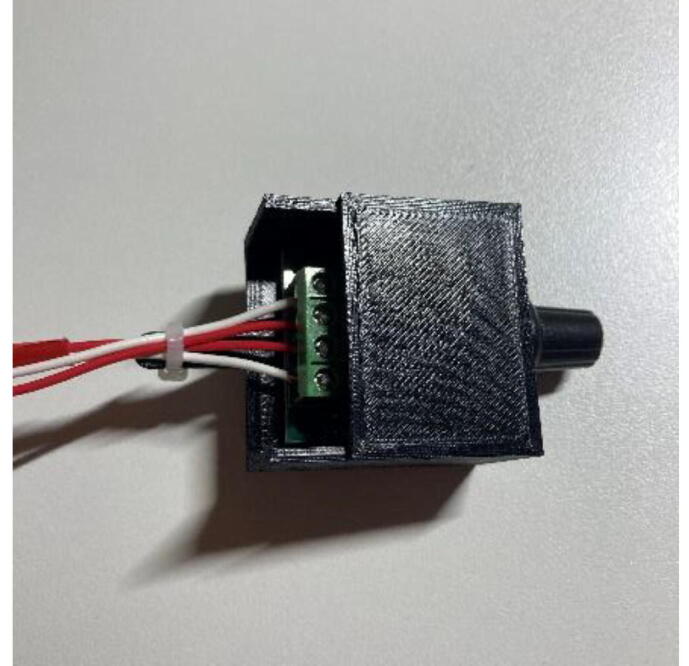
Strain-relief for the wires and attached knob.

Finally, put the knob onto the potentiometer and strain-relieve the wires by zip-tying them to the tongue on the controller case as shown in Fig. 25. This will help prevent the wires from being pulled loose during normal use.

With the controller case assembled, assembly is nearly complete. Next, finish the housing assembly. This will require the housing top (assembled, with the controller attached), the 3-D printed housing bottom, the 60 mm and 105 mm strips of gasket/weather stripping, the M3 × 12 mm screw, a 2.5 mm Allen key (or whatever driver fits the M3 screw in use), and hot glue.

**Fig. 26:**
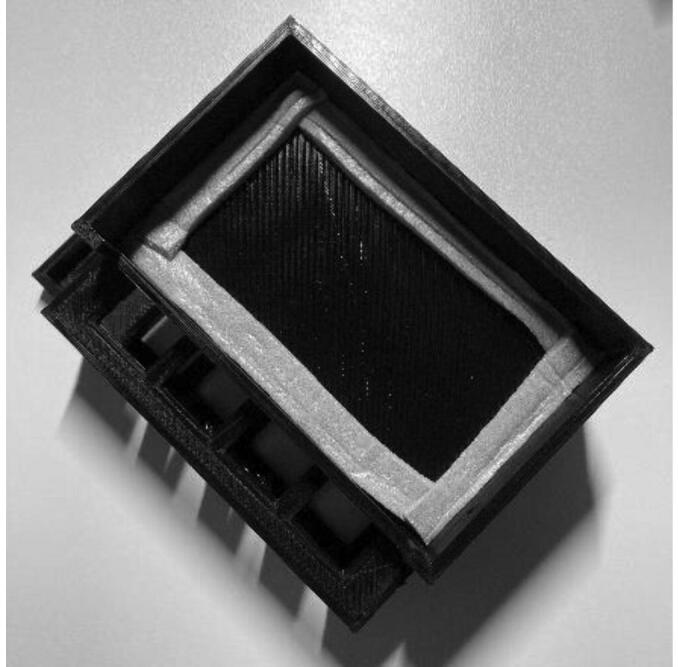
Apply gasket to the perimeter of the housing bottom.

First, apply the weather stripping to the inside of the housing bottom along the perimeter of the flat surface where the housing top fits in (Fig. 26). This will form a seal along the bottom surface of the housing top, preventing unfiltered air from entering the housing.

**Fig. 27:**
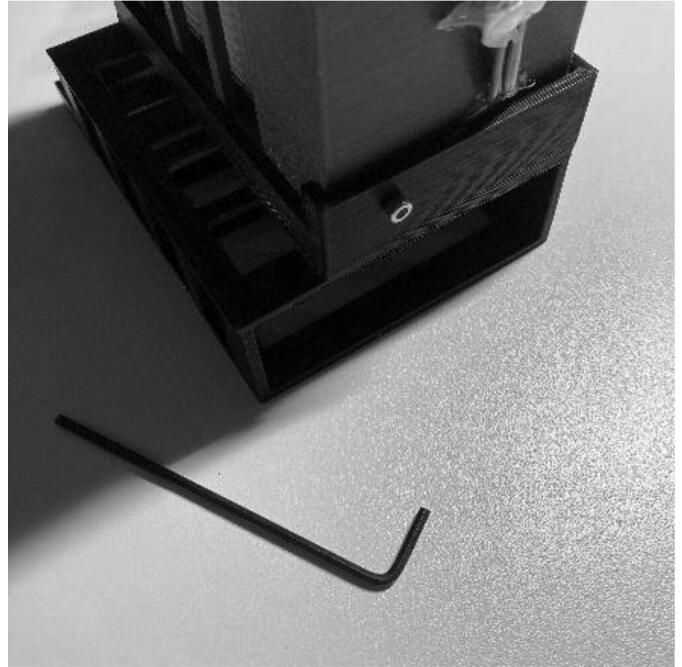
Use the screw to secure the housing together.

With the gasket applied, it is time to assemble the housing. Slide the housing top into the housing bottom, making sure that the fan wires fit into the slot on the side of the housing bottom and that the housing top sits all the way down, compressing the gasket. There is a hole on the side of the housing bottom near the slot for the fan wires. It should be tight enough that the M3 screw threads into the plastic and should align with a similar hole on the side of the housing top. Tighten the screw into the hole using the 2.5 mm Allen key (or applicable driver), securing the housing together, as shown in Fig. 27.

**Fig. 28:**
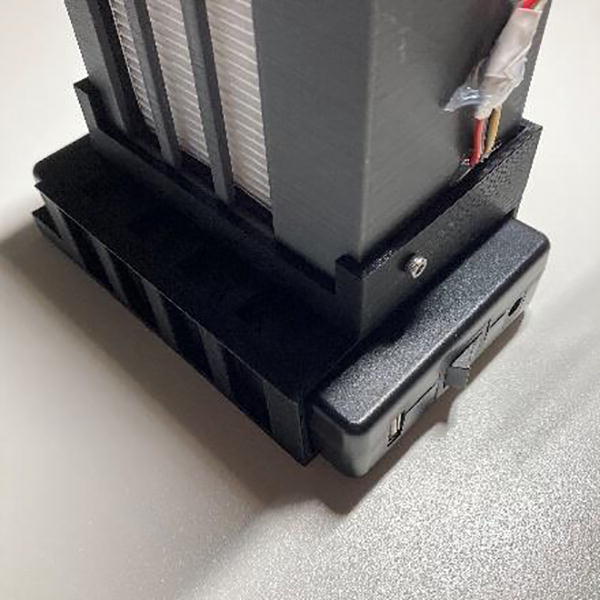
The battery is inserted.

Next, slide the battery into the cage on the bottom of the housing, leaving the power switch and connector on the open side (the same side as the fan wires) as shown in Fig. 28. This placement should leave the battery indicator visible when the battery is on and keeps all wires on one side of the housing. The part is dimensioned so that the battery should have a tight transition fit, meaning it will be held in place by friction, but can be assembled and disassembled by hand. If the battery is not held in place by friction, it can be secured with a rubber band.

**Fig. 29:**
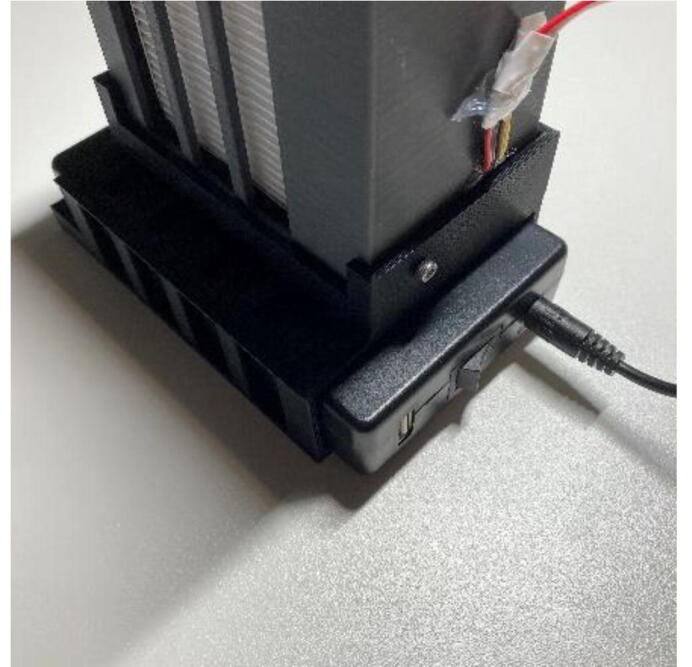
The controller is connected to the battery.

Connect the 12 V power adapter to the battery (Fig. 29). At this point, the fan can be turned on and off by a combination of the switch on the battery and the potentiometer on the PWM controller.

**Fig. 30:**
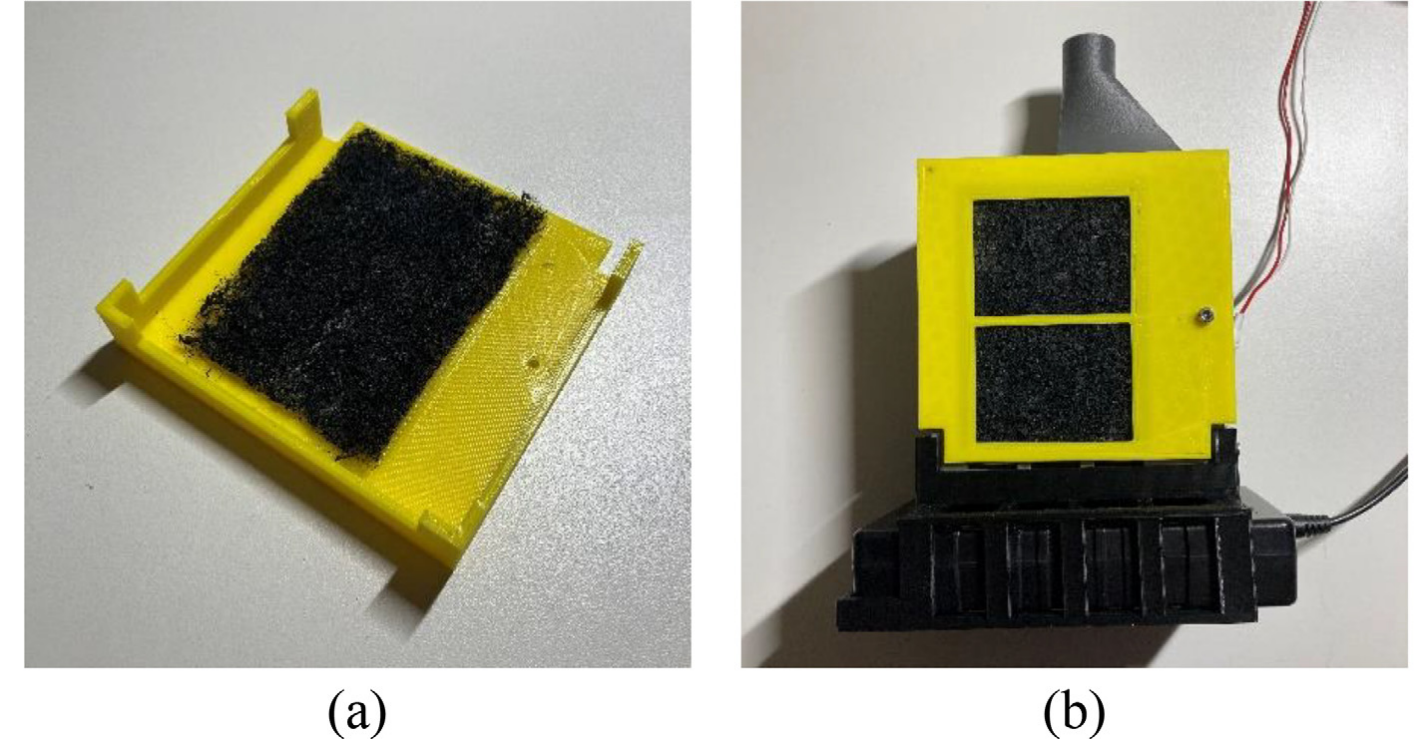
Pre-filter installation (a) fit into cover; (b) install on housing.

If a pre-filter is desired (to help extend the life of the HEPA filter by blocking large particulates), it may be installed on the housing with the pre-filter cover. The pre-filter is intended to be regularly replaced, so the pre-filter cover is designed to be attached to a fully assembled housing with a single M3 screw. Set the pre-filter in the pre-filter cover (Fig. 30a), then install the cover on the housing (Fig. 30b). Do not place a pre-filter inside the housing with the HEPA filter as it could interfere with the seal of the gasket on the filter.

This completes the assembly of the housing. At this point, all parts are ready for use.

## 6 Operation instructions

Donning and doffing procedures should be determined by the local health expert to ensure proper sanitation and disease prevention practices are followed. Published guidelines from 3 M and other sources can be used for reference and the level of risk to health must be considered for all procedures [88], [89], [90].

### 6.1 Donning

**Fig. 31:**
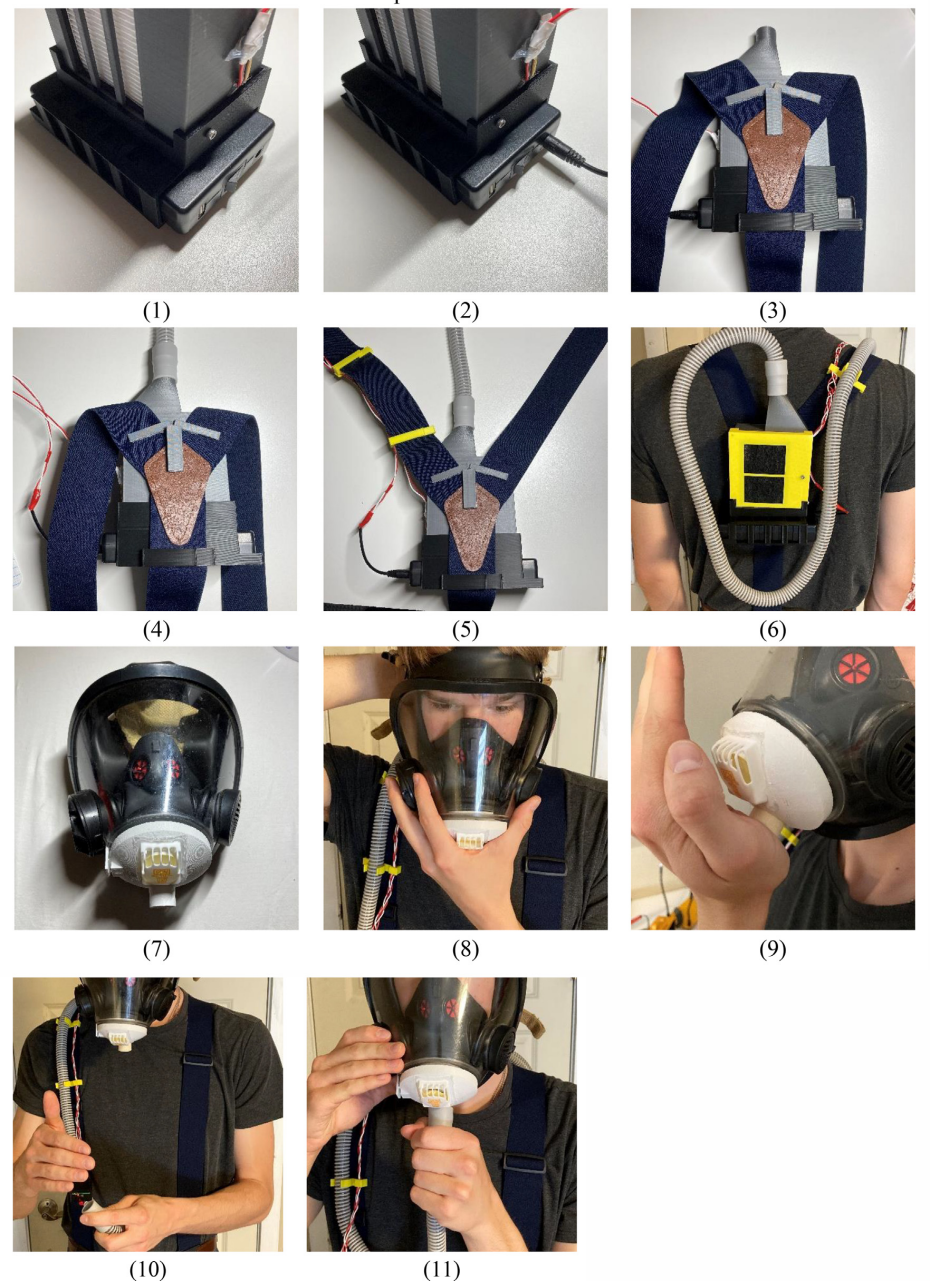
Donning Steps.

The PAPR should be running whenever it is worn. The design and validation of an airflow tester has been left to future work. Such a component would give a quick visual indication of airflow prior to donning the PAPR. This would emulate devices used in 3 M PAPR systems, which are simple objects that use drag forces and air velocity to indicate sufficient airflow [91], [92]. In the design shown here the device enables breathing even in the case of failure – breathing is possible when the fan is off, for example in the case of the battery running out of charge. Using the PAPR in this way is not recommended, as the lack of forced airflow and the presence of a long hose can cause rebreathing of exhaled air, which can be dangerous [75]. The recommended order of operations for donning PAPR is listed here and illustrated in Fig. 31. Videos on donning procedures are available from several sources online [90], [93]. **Note that these steps must be supplemented with disinfecting steps as determined by a local health expert.**

1. Insert a fully charged 12 V battery into the battery cage on the housing.
2. Plug the 12 V connector into the battery.
3. Mount the housing on suspenders.
4. Connect the CPAP hose to the housing.
5. Attach the suspender clips along the desired shoulder, being sure that the hose will not restrict head or arm movement.
6. Put on the suspenders so the PAPR housing sits between the shoulder blades of the wearer. The hose and controller should lay over one shoulder of the wearer.
7. Fit the respirator into the Scott Safety AV3000 mask so it is sealed and locks in place.
8. Don the Scott Safety AV3000 mask.
9. Cover the air inlet and inhale to check for a good seal – the mask should suction to the wearer’s face.
10. Turn on the PAPR, checking for airflow.
11. Connect the CPAP hose to the respirator.

### 6.2 Doffing

Doffing behavior should be similar to donning, ensuring that the PAPR is removed prior to turning off the blower. A local health expert should prescribe necessary disinfecting procedures to be followed during doffing. Videos from online sources may be used for reference [94].

After use, remove the battery from the housing for charging. Connect the battery to the charger provided with the battery. Be sure to keep the battery well ventilated, and on a non-insulating/flammable surface. TalentCell recommends charging their batteries at least every three months [52]. Testing has shown that the indicator lights on the battery included in this BOM **do not** provide any reliable indication of the battery life remaining (details below).

### 6.3 Cleaning and disinfecting

The PAPR should be disinfected according to local, WHO, and CDC guidelines in a manner determined by a local health expert. Resources previously noted, as well as 3 M’s guidelines for cleaning and disinfecting PAPR assemblies should be used for reference [89]. The PLA used in the housing is chemically compatible with most standard cleaning agents [95]. PPE such as nitrile gloves should be worn while disinfecting the equipment. All equipment should be cleaned according to a risk-based assessment of the situation. Purchased components such as the CPAP hose should be cleaned according to any instructions provided with the component in order to avoid damaging the components. Regular replacement of the HEPA filter and its pre-filter are recommended – 3 M suggests replacement after each use and recommends against attempts to clean the filter [89].

**Fig. 32:**
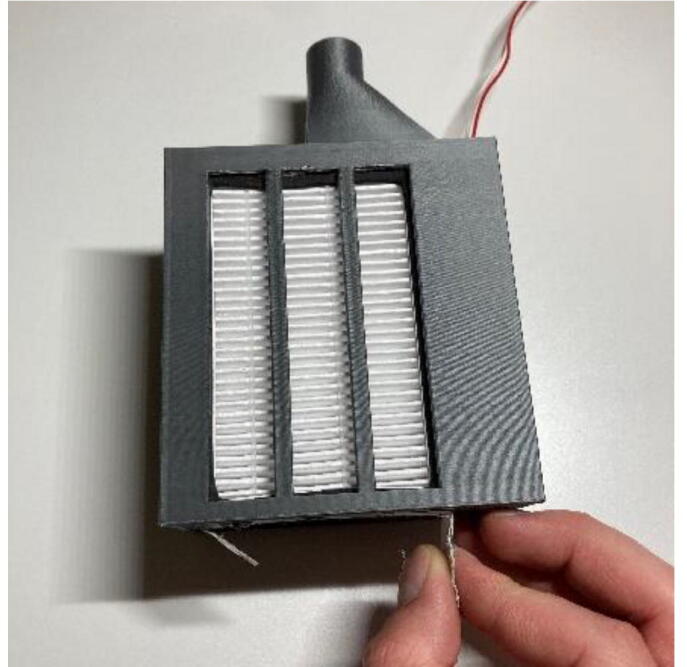
Use tabs to remove HEPA filter.

In order to replace the HEPA filter, use a 2.5 mm Allen key (or the appropriate driver for the screw used) to loosen the M3 screw holding the housing together. Separate the housing top and bottom. Grasp the duct tape tabs exposed on the bottom of the housing top. Pressing against the case, pull the filter out of the housing as shown in Fig. 32.

In order to facilitate future filter changes, be sure to apply a duct tape strip in the manner shown in Fig. 11 prior to installing the new HEPA filter.

## 7 Validation and characterization

In order to validate this design, it was evaluated against NIOSH standards for PAPR airflow as defined in 42 CFR 84 subpart K [71]. Note that an airflow test is one of many tests required to certify a PAPR for use, particularly in a CBRN environment [96]. A battery life test was also completed in order to verify that the PAPR can provide sufficient airflow for the full length of time that it is expected to be worn.

The testing completed here shows that the components used in this design have the mechanical capability of meeting NIOSH requirements (airflow and service life). Other components and design details were selected with the intention of bringing this device as close to NIOSH certifiable as possible. However, for this PAPR to be used or sold commercially, more testing must be completed to prove that it meets all requirements specified in 42 CFR 84 subpart K (this is discussed further below). A comprehensive list of testing procedures is available from the CDC’s NIOSH site [96].

### 7.1 Flow rate

NIOSH specifies that the blower on a tight-fitting PAPR must provide 115 L per minute of air when installed in the housing. Airflow can be measured in several ways, such as with a mass-air-flow sensor (MAF) or by measuring the differential pressure across a known resistance (e.g. an orifice plate). Since the airflow rate is low and the fan is sensitive to airflow resistance, the preferred method for this case would be to use a MAF sensor.

**Fig. 33:**
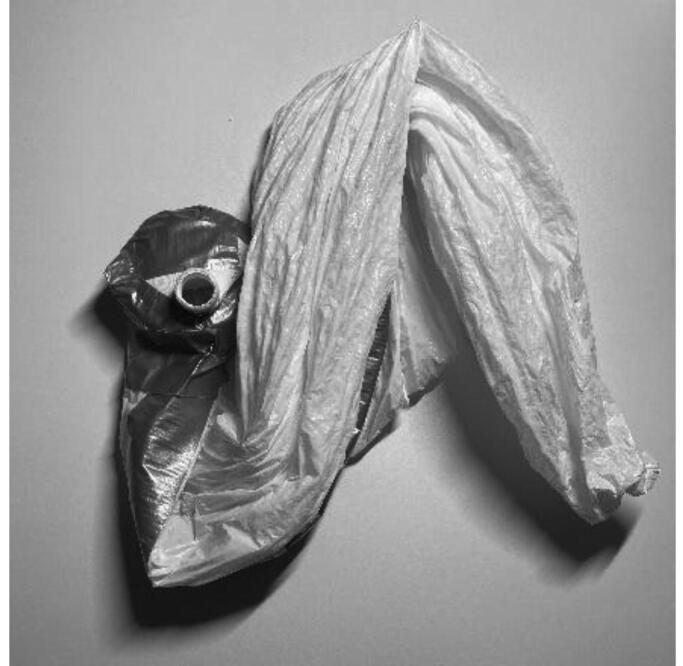
Airflow testing bag.

Here the airflow rate was measured in a more accessible manner – by using the PAPR to fill a known volume. The respirator was sealed into the opening of a polyethylene bag with a manufacturer-listed volume of 49.2 L. The exhalation valve was sealed, as was the rest of the bag, leaving the CPAP connection point (the air inlet) as the only path for air to enter or leave the bag. The bag is pictured in Fig. 33 with the air pressed out.

**Fig. 34:**
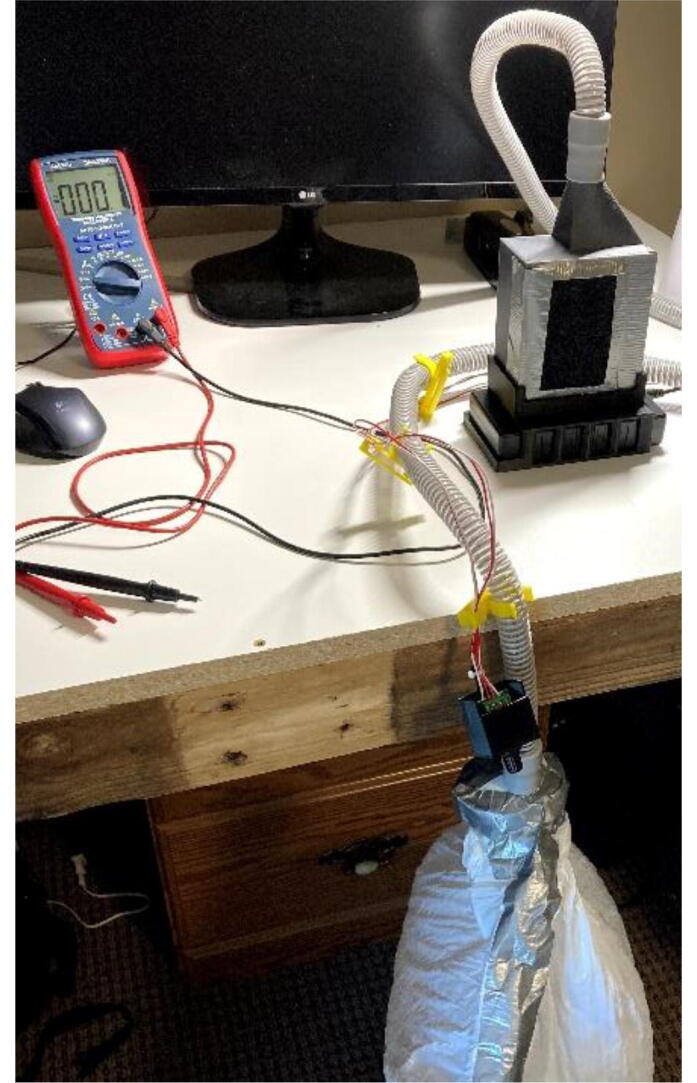
The airflow test setup, mid-test.

Prior to connecting the PAPR to the bag, all air possible was squeezed out of the bag (a vacuum was not used). The PWM controller was set, and the average voltage applied to the blower was measured. Then, the bag was clutched so as to block airflow into the bag. The CPAP hose was attached to the fitting on the bag. A stopwatch was started as the bag was released, allowing air to flow into the bag. The fill was timed from the release of the bag until it became taut (indicating it was ‘full’ with air). The test setup is shown in Fig. 34.

The measured time was used to estimate airflow, *Q,* by dividing the volume of the bag, *V*, by the fill time in minutes, *t,* in the equation *Q = V/t*.

This approach comes with inherent error, which should be considered. Errors that could make the airflow seem higher than it is were corrected for, while errors that could make airflow seem lower than it actually is were acknowledged, but not corrected for. The intention in this approach is to acknowledge that these measurements are imprecise and to attempt to underestimate the total airflow. This conservative approach ensures that the PAPR has a minimum air flow as tested here.

The bag was not entirely devoid of air when the test is started (a vacuum was not used). The exact volume of the bag is also unknown due to how it was sealed, and that the manufacturer does not clarify how the volume is initially measured. To account for this, the volume used in all calculations is 48 L instead of the 49.2 L specified by the manufacturer.

As the bag approaches its full volume, back pressure increases as air tends to flow back out of the bag, inducing leakage from the bag. This back pressure is also known to decrease the flow rate of the fan [69]. Since these should both decrease the calculated flow rate, they are not corrected for in any calculations.

**Fig. 35:**
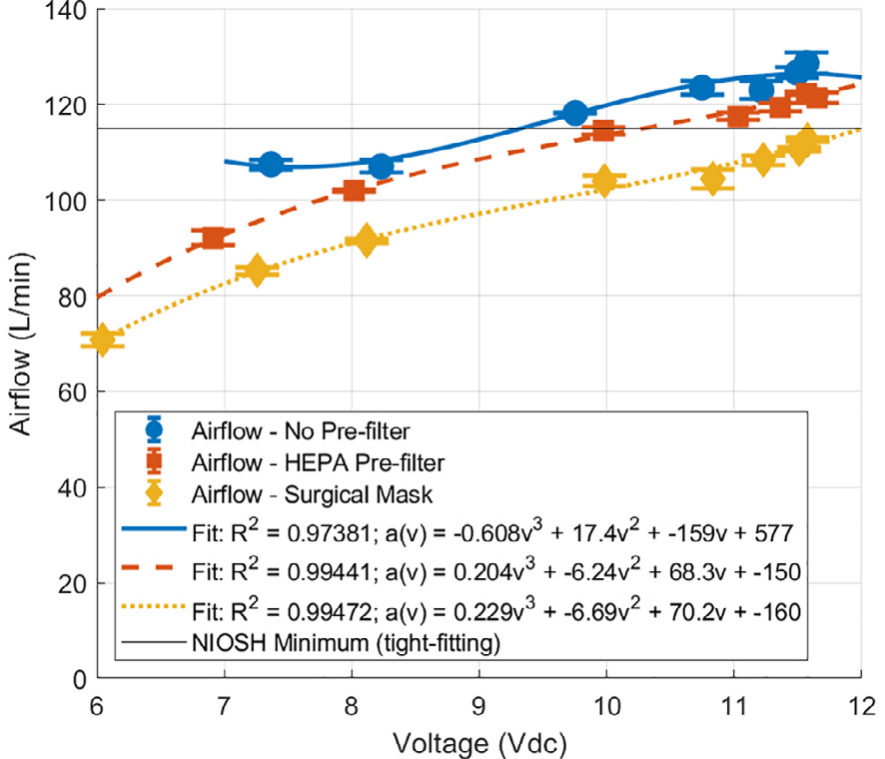
PAPR airflow test results.

Tests were run for 10 equally spaced potentiometer positions on the PWM controller, corresponding to 10 average voltages applied to the fan. At low voltages (the first two or three knob positions) the fan did not turn on. The RMS voltage of the duty cycle was measured using an AstroAI DM6000AR True-RMS multimeter [97]. These tests were conducted with no pre-filter, a HEPA pre-filter, and a surgical mask pre-filter. Each test was conducted 3 times, providing an opportunity to check the repeatability of measurements. The average measured airflow for each test case is shown in Fig. 35. The average standard deviation amongst all data points was 1 L/min, with the maximum standard deviation being 2.25 L/min. Error bars on the gathered data points indicate a single standard deviation above and below the average value. The data points were approximated with a 3rd order polynomial curve fit using a linear least squares method. The data points were weighted by the inverse of their standard deviation to aid the fit – this was observed to increase R-squared for all three fits.

As can be seen in Fig. 35, without a pre-filter of any kind, the PAPR provides sufficient airflow for any fan voltage greater than 9.5 V. With a HEPA pre-filter, it is able to provide sufficient airflow for a fan voltage greater than around 10.5 V. When using a surgical mask pre-filter, the airflow is never sufficient to satisfy NIOSH requirements, meaning it should not be used in a commercial setting. The full dataset is available in the OSF under *data\Testing.xlsx* [43].

### 7.2 Battery life

Battery life limits the service life [98] of the product. To test the battery life, the battery was fully charged and the PAPR was fully assembled (connected to the AV3000 facepiece (mask) with a CPAP hose). The mask was sealed with cling wrap to simulate a person wearing the mask. This left all air to exit through the exhalation valve, as would be the case during normal use. The PAPR was turned on with the PWM controller set to 100%, as this is expected to be the case with maximum current-draw. The voltage of the battery at the input terminals on the PWM controller was measured every 15 min until the battery died (the blower turned off). This was tested with no pre-filter and with a surgical mask pre-filter installed, offering the lowest and highest resistive loads for the blower.

When running the PAPR at 100% duty cycle, starting with a fully charged 6000 mAh battery, the PAPR ran for over 7 h in both cases. The expected use time, according to Greg Ball from the Oakland Township Fire Department in Michigan, is around 70 min. This means that airflow will continue in some capacity for any expected use of this PAPR. However, the voltage of a rechargeable battery decreases as it is used. This battery’s voltage dropped from a starting voltage of around 12.2 V to around 9 V by the time the battery died. This voltage decrease has an effect on the airflow as shown in Fig. 35.

**Fig. 36:**
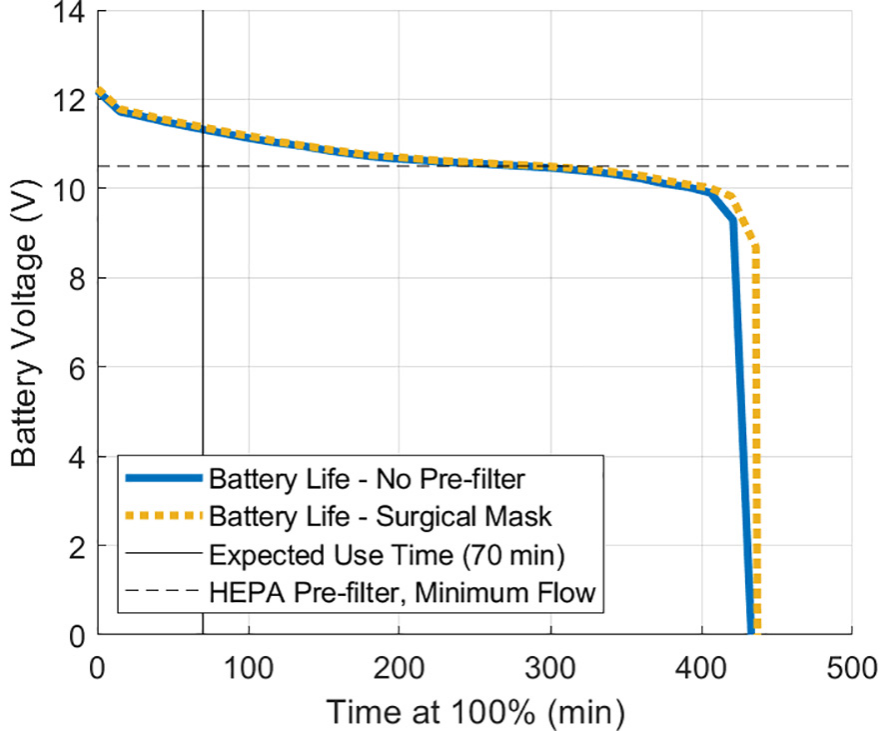
Battery voltage as a function of run time with no pre-filter and with a surgical mask prefilter.

In order to assess how long the PAPR can provide sufficient airflow according to NIOSH standards, the battery voltage over time was plotted, then overlaid with the expected use time and the approximate voltage at which airflow drops below 115 L/min with a HEPA pre-filter. This is shown in Fig. 36, which indicates that airflow will drop below the NIOSH standard after around 280 min of use, or 4 h and 40 min. This is 400% of the expected use time (calculated by dividing $\frac{\mathrm{actuallife}}{\mathrm{expecteduse}}$).

**Fig. 37:**
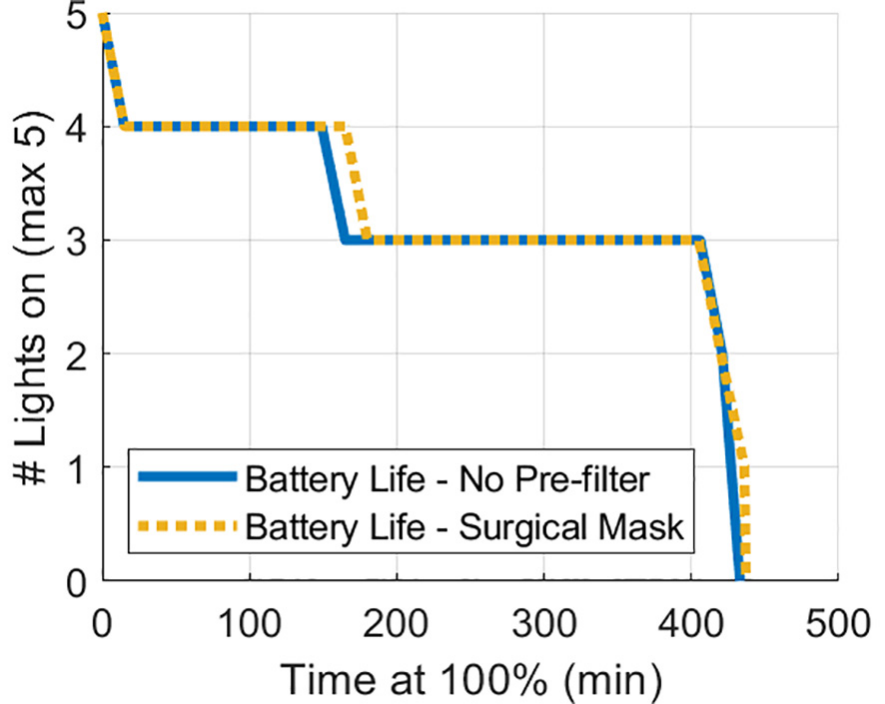
Battery life indicator lights.

This testing suggests that stepping down to a 3000 mAh battery could be a reasonable design change to save on cost and weight, although this has not been tested. An important detail that was discovered during testing is that the indicator lights on this particular battery do *not* give a good idea of the remaining life of the battery. As can be seen in Fig. 37, three of five lights on could indicate a remaining battery life anywhere from 20 min to over 4 h (250 min). As such, it is highly recommended that the battery simply be brought to a full charge after each use, while following instructions provided with the battery on charging and maintaining the battery.

## 8 Safety

Neither the safety nor the effectiveness of this product is certified by NIOSH, 3 M, or Michigan Tech. This product has been shown to provide sufficient airflow to allow the PAPR to meet NIOSH requirements, and it is built with components that were selected with NIOSH certifiability as a high priority. It has been qualitatively tested to show that breathing is possible even without the blower running and the hose was selected and constrained in the design in order to prevent kinking or blockages in the hose. This means that suffocation due to malfunction of the device is highly unlikely, provided that it is used in the expected manner. This device is not intended to be used without the blower on, as extended use has the potential to cause CO_2_ rebreathing, which can be harmful to one’s health [75].

The HEPA filter used in this design does not contain fiberglass, according to Sears Parts Direct [99]. Regardless of this, Smart Air Filters, a manufacturer of filters, has noted that experimentation has shown with some confidence that any fibers shed by a HEPA filter fall far below requirements set by NIOSH and WHO and should not be harmful to respiratory health [100].

In order to produce this device commercially, further development and testing is required in order to receive NIOSH approval for use as a CBRN PAPR [101], which is the intended use of this design. There are two classes of PAPR defined by the NIOSH standard: HE and PAPR100. Each of these has different but overlapping requirements listed in 42 CFR 84 subpart K. PAPR100 devices have been recently introduced, removing some old requirements while adding new features. Both have requirements for airflow resistance, fitment, filter efficiency, noise level, and breath response. HE devices must sustain silica dust loading tests, while PAPR100 devices must undergo particulate loading tests and communication performance tests. PAPR100 devices must also have a low-flow warning device [71]. To meet CBRN requirements from the CDC, PAPRs (both classes) must also undergo tests from other sections of 42 CFR 84, which challenge the longevity of the PAPR against physical and chemical abuse [73].

This study is intended to outline and detail the design process for an open source device, to provide a framework on which to build such a device, and to provide a low-cost, accessible option for PPE in such an event as the current COVID-19 pandemic. Distributed manufacturing of open hardware has been shown to be an effective means to meet demand for a wide array of medical supplies during this immense supply shortages observed in the current pandemic [102], [103], [104], [105], [106], [107], [108], [109], [110], [111].

Safety procedures for the use (donning and doffing) and cleaning of this equipment should be determined by local health personnel. 3 M and other sources provide a wide array of resources for identifying and developing safety and disinfecting procedures as previously discussed.

## 9 Discussion

### 9.1 Function

Although PAPRs are effective for COVID-19 and other airborne pathogens as long as an appropriate filter is used and will thus be useful to firefighters and other first responders in this and future respiratory-virus pandemics, PAPRs should not be used for firefighting or in oxygen-deficient environments [NIOSH]. For those the full SCBA systems should be used.

### 9.2 Economics

The total cost of this conversion kit is assessed assuming the tools and Scott Safety AV3000 facepiece are already owned. The total cost of a single PAPR, corrected for the amount of material used (e.g. the cost of the length of wire used, rather than the cost of a whole spool of wire) is $146.09. Production of a single unit will cost more than this, due to the initial investment required for some components/materials.

The majority of this cost is incurred by the battery and the fan. Further testing could be conducted to assess the use of lower cost components, but there are limitations on both the battery and the fan when reducing cost. The battery has been shown to last far longer than the expected use time for the PAPR, but the voltage drop during use is significant enough that airflow can fall below NIOSH requirements during extended use. Similarly, a lower cost fan will most likely be unable to meet the static pressure requirements of this system. This is a greater limitation, as fan output can drop precipitously as the static pressure limits are reached [69].

This PAPR is an open-source approximation of the Scott Safety C420 Plus PAPR [112]. Note that this device has not been approved or supported by 3 M in any significant way at this time. According to Federal Resources [113]:

The price would be $975.82 for the C420 PAPR Kit p/n 200833–30. This includes Blower, LiSO2 Battery, Decon Belt, 30″ CBRN AV- PAPR Hose.

The cost savings between this open source PAPR conversion kit and the Scott Safety C420 can be calculated $\frac{\mathrm{commercial}-OS}{\mathrm{commercial}}$*.* The savings here are 84.8%. It is worth noting that the cost of the Scott Safety C420 includes testing and NIOSH certification that this open source PAPR design lacks, but could be added in future work for those wanting to commercialize this open source design. It should be noted that manufacturers of the core components (e.g. 3 M) could use the specialty component supplier business model to encourage commercialization of the open hardware and increased demand for their components [114], while reducing costs for their customers.

Thanks to the assumption of a pre-existing SCBA mask, this PAPR has the added advantage of being much less expensive than other PAPR systems. For example, the 3 M Versaflo TR-600 PAPR is listed on Fisher Scientific for $1,800.36, yielding a savings of 91.7% [115]. It can also be converted back to a SCBA.

### 9.3 Future work

This open source design provided here provides a framework for the development of a commercial device. As discussed in Section 8, more testing and feature additions are required to meet NIOSH standards. Testing would include airflow resistance testing, chemical resistance testing, filter service life testing (HEPA filters only filter out certain chemicals for so long), and breath response testing. These requirements are listed rather concisely in 42 CFR 84 subpart K with detailed testing procedures provided on the CDC website [96]. Some additional testing is required for CBRN certification. NIOSH has a document published on the CDC Stacks that explains and puts into perspective extra testing required for CBRN PAPRs [116]. Feature additions may include a low-flow indicator and a low-battery indicator that alert the wearer that they must move to a safe area and resolve the issue. Airflow detection could be introduced in a few ways, briefly explored here:

• *Voltage sensing* – data could be gathered to generate a more robust version of the curves in Fig. 36 which could be used as a lookup table to estimate airflow based on battery voltage alone. This approach is fraught with assumptions and would not remain accurate over time. Voltage sensing is more useful for battery-life indication.
• *Voltage and current sensing* – airflow and blower rotation speed are directly related. This is true in theory and observations. The relationship between motor speed and voltage and current is also well defined in theory. Tests could be completed to characterize the blower, allowing estimation of airflow from voltage and current. This still has inherent error and nonlinearities and will vary from fan to fan.
• *Pressure sensing* – CPAP devices measure airflow using differential pressure across a known resistance, such as a venturi nozzle or orifice plate [117]. The challenge with this approach is that it requires a resistance be added to the system (unless the housing nozzle were redesigned into a venturi), which could reduce airflow.
• *MAF sensor* – low-cost hot film anemometers are accessible and can be used to measure airflow from the cooling effect on a heated film [118], [119]. This offers the most direct and low-impact measurement of airflow [120].

There are other design modifications which would positively affect the usability, accessibility, and cost of this device. First, an airflow indicator could be designed to indicate sufficient airflow. The expected air velocity can be determined using the diameter of the hose and the required airflow rate. Using this, a part could be designed with a specific mass and drag coefficient such that it floats if the blower is providing sufficient airflow. This would emulate a variety of parts available from 3 M [91], [92].

The greatest cost for this device comes from two components – the blower and the battery. Both of these could be replaced with distributed manufacturable devices. It has been shown that batteries can be built safely in DIY settings, although such devices can prove to be heavy [121]. A rechargeable battery could also be constructed from individual cells, with charging hardware (e.g. overcharge protection [122]) and the housing built around it. 3-D printable, open source centrifugal blowers have also been designed in the past [123]. Such a blower could be built and tuned to meet the airflow and pressure requirements of this device, reducing cost and potentially the weight of the device.

The cost of the 3-D printed components was $9.10 (or about 6% of the total); this cost could be further reduced to about 1% of the total by using several paths of distributed recycling and additive manufacturing (DRAM) [124], [125], [126], [127], [128], by either using a recyclebot to make filament [129], [130] or direct fused particle fabrication (FPF) or fused granular fabrication (FGF) [131], [132], [133]. Future work is needed to assess the cost savings for DRAM as well as using these open source designs with other brands of base components.

## 10 Conclusions

This study showed that an open source PAPR conversion of a SCBA can be fabricated with a low-cost 3-D printing and widely available components for less than $150. This low-cost conversion saves 85% on commercial conversion kits and over 90% for proprietary PAPRs. In this study it was demonstrated with a 3 M SCBA, but the parametric designs allow for adaptation to other core components and can custom fit specifically to fire-fighter equipment, including their suspenders. The open source PAPR has controllable air flow and enables breathing even if fan is disconnected or battery dies. The open source PAPR was tested for air flow as a function of battery life and was found to meet NIOSH air flow requirements for 4 h and 40 min, which is 300% over regular use. It is expected that the design laid out in this study does or is close to meeting NIOSH requirements. To commercialize this device, however, further testing is necessary to validate that the PAPR can withstand environmental stressors to meet requirements from NIOSH and the CDC.

## Declaration of Competing Interest

The authors declare that they have no known competing financial interests or personal relationships that could have appeared to influence the work reported in this paper.
